# Functional Roles Affect Diversity-Succession Relationships for Boreal Beetles

**DOI:** 10.1371/journal.pone.0072764

**Published:** 2013-08-20

**Authors:** Heloise Gibb, Therese Johansson, Fredrik Stenbacka, Joakim Hjältén

**Affiliations:** 1 Department of Zoology, La Trobe University, Melbourne, Victoria, Australia; 2 Department of Wildlife, Fish and Environmental Studies, Swedish University of Agricultural Sciences, Umeå, Sweden; University of Waikato (National Institute of Water and Atmospheric Research), New Zealand

## Abstract

Species diversity commonly increases with succession and this relationship is an important justification for conserving large areas of old-growth habitats. However, species with different ecological roles respond differently to succession. We examined the relationship between a range of diversity measures and time since disturbance for boreal forest beetles collected over a 285 year forest chronosequence. We compared responses of “functional” groups related to threat status, dependence on dead wood habitats, diet and the type of trap in which they were collected (indicative of the breadth of ecologies of species). We examined fits of commonly used rank-abundance models for each age class and traditional and derived diversity indices. Rank abundance distributions were closest to the Zipf-Mandelbrot distribution, suggesting little role for competition in structuring most assemblages. Diversity measures for most functional groups increased with succession, but differences in slopes were common. Evenness declined with succession; more so for red-listed species than common species. Saproxylic species increased in diversity with succession while non-saproxylic species did not. Slopes for fungivores were steeper than other diet groups, while detritivores were not strongly affected by succession. Species trapped using emergence traps (log specialists) responded more weakly to succession than those trapped using flight intercept traps (representing a broader set of ecologies). Species associated with microhabitats that accumulate with succession (fungi and dead wood) thus showed the strongest diversity responses to succession. These clear differences between functional group responses to forest succession should be considered in planning landscapes for optimum conservation value, particularly functional resilience.

## Introduction

An increase in species diversity with forest age has been observed in many ecosystems [1–3] and is an important motivation for the conservation of significant areas of long-undisturbed habitats [4]. Early successional stages support a unique disturbance-associated fauna, with anthropogenic disturbances, such as clear-cutting, having similar effects to natural disturbances such as fire if managed appropriately [5,6]. Species associated with later successional stages are expected to accumulate over time as a result of colonisation events and the accretion of new habitats, which provide a greater diversity of resources [7]. In regions where late successional stages previously dominated the landscape, e.g. boreal forests [8,9],, we might expect that these habitats would support more species than more recently disturbed habitats, which historically occupied a smaller area. This could result purely as a consequence of the species-area relationship [10].

Although positive diversity-age relationships are common, the relationship between species diversity and succession may differ between guilds or functional groups. This is expected as different species are dependent on resources that peak at different points in succession. In particular, species that are tied to microhabitats, such as dead wood, that tend to increase in availability and diversity with forest age may benefit more from succession than those that rely on succession-independent microhabitats. Functional roles may thus be a key determinant of diversity responses to habitat succession. Knowledge of how responses differ among species with different functional roles is critical in determining the resilience of ecosystems and specific functions to changes in disturbance regimes. For example, we expect species important in the decomposition of dead wood to be more sensitive to succession than generalised predators, which has consequences for the sustainability of those functions [5].

Although the effects of succession and disturbance on species composition are well established [11–13], the highly specific habitat use of many species means that more broadly applicable indices of diversity are important in comparing patterns across ecosystems and biomes. Identity-free diversity is commonly measured using a range of simple to complex measures. Simple indices include species richness and abundance; more derived indices include Shannon’s diversity index (H) [14] or Pielou’s evenness index (J) [15]; and models of rank-abundance relationships explore the structure of assemblages in more detail. A variety of different rank-abundance models have been proposed, with a diversity of possible mechanisms producing each curve. The best model is thought to depend on successional stage [16,17], environmental severity or homogeneity [18,19], species richness [20] or taxonomic breadth [19]. The most desirable of these models include parameters that can be considered to be biologically meaningful. The model that best fits a data set and its parameters might be expected to change with habitat succession. For example, if the importance of competition increases at later successional stages, as proposed in the intermediate disturbance hypothesis [21,22], we might expect models representing competitive mechanisms to increasingly appear as the best descriptor of assemblage structure as a habitat ages. We might also expect that the importance of competition will depend on the functional groups examined and how easily their preferred resources might be dominated.

Beetles are an inordinately diverse animal group (J.B.S. Haldane, quoted by Hutchinson 1959 [23,24] [24,25]) and beetle assemblages therefore present an ideal model system in which to test the effect of functional roles on diversity responses to succession. An improved understanding of beetle diversity responses to succession will provide insights into the resilience of diverse functional groups to habitat disturbance resulting from anthropogenic land use and the increase in natural storm and fire events associated with climate change. We used beetle assemblages in Swedish boreal forests, where harvesting regimes have led to significant reductions in the area of older stands and many beetle species are red-listed [26], to address the following questions:

1 Which rank-abundance model is the best fit to the rank abundance curve and does the identity of the best-fit model depend on time since disturbance or beetle functional group? We predicted that models consistent with competitive structuring of a community would provide a better fit to the data at later successional stages, as suggested by the intermediate disturbance hypothesis. We considered “functional” groups based on: a) threat status; b) dependence on dead wood (“saproxylic”); c) diet; and d) trap type, considered indicative of the ecological breadth of the assemblage (here, flight intercept traps collected species with a broad set of ecologies, i.e., any flying species, while emergence traps targeted a narrow set, i.e., species feeding on dead wood). We expected that functional groups reliant on a narrower diversity of resources would include more species that compete directly and would therefore be more likely to be competitively structured. For example, beetle assemblages collected on relatively uniform supplemented logs are expected to show greater competitive structuring than those collected using a less specific trapping method such as flight intercept traps.
2 Does diversity increase with time since disturbance and does this relationship depend on functional group? Here, we test the relationship between stand age and diversity for the functional groups considered in question one. We include simple measures of diversity such as abundance and species richness, traditional diversity indices and model-derived diversity indices.

We expected: a) threatened (red-listed) species would increase in diversity with stand age more rapidly than common species as species are commonly threatened through loss of habitat; b) after an early peak due to high dead wood availability after clear-cutting, specialists on dead wood would increase more rapidly with stand age than other species as more suitable and diverse habitat becomes available; c) species that feed on fungi and detritus, which slowly accumulate through succession, would increase more rapidly than predators; and d) assemblages of species collected using emergence traps would increase in diversity more slowly with succession (because the total pool of specialist species would be smaller) and be more competitively structured (because they rely on a single type of resource) than those collected using flight intercept traps.

## Methods

### Ethics statement

All necessary permits were obtained for the described field studies. Permits were obtained from Västerbotten Länstyrelsen, Sweden, and the forest companies Holmenskog, Sveaskog, and SCA allowed access to their land.

### Study sites

We worked in the central-boreal vegetation zone of Sweden [27] between the latitudes 63.6 N and 64.3 N and longitudes 16.9 E and 20.1 E, and altitudes from 100 to 550 m a.s.l. Stands in the study areas were Norway spruce-dominated (*Picea abies* Karst.) forests of *Myrtillus*-type understory [28], mainly surrounded by managed forests of a range of age classes. Scots pine ( *Pinus* *sylvestris* L.) was the next-most common tree species, while birch ( *Betula* spp.) and aspen ( *Populus* *tremula* L.) also occurred in sparse populations, both in the research areas and surrounding forests. We selected nine areas that included five stands of spruce within a 15 km radius, with each stand representing one of five age classes: 1) old-growth stands in or in direct association with a nature reserve or national park (mean tree age 160 yr, mean stand size 249 ha); 2) mature production stands (120 yr, 10 ha); 3) middle-aged, recently commercially thinned stands (53 yr, 8 ha); 4) young unthinned forest stands (30 yr, 16 ha); and 5) clear-cut areas (5–7 yr, 16 ha) (for further information, see Stenbacka [29] et al. 2010). This set up allowed us a broad spread of stand ages and the blocked design prevented problems with spatial autocorrelation.

### Insect sampling and identification

Beetles were sampled continuously, i.e., traps were active, from May to September 2006 using three flight intercept traps at each of the forty-five sites. During this period, emergence traps [30–33] were placed on spruce logs (4 m by 200-250 mm diameter (ø)), which had been placed out in the old-growth, mature and clear-cut sites (described as age classes 1, 2 & 5 above; n=9 for each class) in winter 2001-2002. Logs were separated by approximately 50 m. The emergence traps were designed to collect all insects emerging from a 30 cm enclosed section of the logs by wrapping this section in polypropylene weed barrier cloth, which was kept separated from the log by wire. The traps were sealed with wire at both ends and moved to a new position on the log every trapping year to avoid any influence on natural colonization or succession by insects. Emerging insects were collected in a white 300 ml plastic bottle, attached to the top of the trap and one-third filled with 50% propylene glycol and some detergent to reduce the surface tension.

We placed three flight-intercept traps (Polish IBL2-traps; CHEMIPAN, Warszawa, Poland; see Pettersson et al. [29] 2007) 50 m apart in each stand. The intercepts were directed toward 0^°^, 120^°^, and 240^°^. The traps were hung on a polypropylene rope (ø 6–8 mm) strained between two trees, or in some cases on clear-cuts between wooden poles (ø 27 mm and 2.5 m long). Additional cords were strained to the ground to make the traps less wind sensitive. Beetles were collected in 600 ml plastic bottles one-third filled with 50% propylene glycol with a small amount of detergent. A rainwater drainage module was attached between the trap and the bottle to avoid overfilling of the bottle and dilution of the glycol solution.

All collected beetles were identified to species, with the exception of *Acrotrichis* sp. (Family Ptiliidae). We used Speight’s [34] definition of saproxylic and also classified saproxylic beetles according to feeding habits (cambium consumers, detritivores, fungivores and predators) using The Saproxylic Database (Nordic saproxylic network, 2010, www.saproxylic.org), to which species confined to the northern part of Sweden were added (Hilszczański, J, Pettersson, R. and Lundberg, S. pers. comm.) (Appendix S1). The beetles were classified as red-listed based on the Swedish red list [35]. Nomenclature and taxonomy of the beetles follows [36,37]. We used those species that fit distinctly within each category and did not include generalists, e.g., species that consumed both detritus and fungi.

Allocation to functional groups allowed us to determine if the ecological role of species affected the way in which measures of their diversity responded to succession. In particular, we compared species that differed in threat status (red-listed and common species), in their reliance on dead wood (obligatory saproxylic, facultative saproxylic and non-saproxylic), diet (specialist cambium consumers, detritivores, fungivores and predators) and the way in which they were collected (emergence traps on experimental logs, which collect species specifically associated with dead wood of an early decay stage, and flight intercept traps, which collect a broader fauna). For all comparisons, except the trap-type comparison, we used only data collected in flight-intercept traps to encompass all forty-five sites.

### Analyses

For analyses, we used data pooled from all window traps or all emergence traps, such that n = 45. To determine whether stand age affected the form of the beetle species rank abundance distribution, we used the package vegan on R 2.12 [38]. We used Akaike’s information criterion (AIC, Akaike 1974 [39]) to find the best fit model for assemblages collected in each stand from a range of proposed models including purely statistically descriptive models and models representing niche-apportionment, i.e., a competitively structured community [40]. We compared the niche-oriented Broken Stick and Dominance-Preemption models and the descriptive (null) Lognormal, Zipf and Zipf-Mandelbrot models (Table 1). The niche-apportionment models can be interpreted as indicative of the importance of competition in structuring assemblages. However, competition is not necessarily the driver for these patterns and alternative mechanisms can result in similar models [41], so interpretation of causation should be cautious.

**Table 1:** Rank abundance models tested, their authors and formulae.

**Model (Author)**	**Formula**	**Parameters**	**Description**
***Niche-apportionment***			
Broken Stick (MacArthur 1957, Pielou 1975)	*a*_*r*_ * = (J/S) ∑* ^S^_*x=r*_ * (1/x)*	*J* = abundance; *S* = no. species	Represents a resource pool, imagined as a stick, broken by *n-1* points thrown randomly along the stick, representing the niches of *n* species; Can be imagined as a group of *n* species of equal competitive ability simultaneously occupying the total niche and jostling to determine niche boundaries (Tokeshi 1993)
Dominance-Preemption (Tokeshi 1990)	*a*_*r*_ * = Jα(1-α)* ^*(r-1)*^	*J* = abundance; α = decay rate of abundance per rank	Describes the least even species abundance distribution, where, after initial colonisation or speciation, each new species pre-empts more than 50% of the smallest remaining niche
***Descriptive***			
Log-Normal (Preston 1948, 1962)	*a*_*r*_ = *e* ^(log(μ) + log(σ)N)^	*N* = normal deviate; μ = mean log_e_(abundance); σ = standard deviation log_e_(abundance)	Assumes that the logarithmic abundances are distributed normally
Zipf (Zipf 1949)	*a*_*r*_ = *J(*p1*)r* ^γ^	*p1* = fitted proportion of most abundant species; γ = decay coefficient.	Zipf’s law states that the frequency of any species is inversely proportional to its rank.
Zipf-Mandelbrot (Mandelbrot 1965)	*a*_*r*_ = *Jc(r*+ β*)* ^γ^	*c* = meaningless scaling constant; β = deviation below the asymptote described by γ	Using a fractal tree, Mandelbrot generalized the Zipf model to produce the Zipf-Mandelbrot distribution (McGill 2007). Assumes habitat can be considered hierarchical in structure (Barangé & Campos 1991)

*a*_*r*_ represents the expected abundance *a* of of species at rank *r*. Models were either biologically oriented “niche-apportionment” or statistically oriented “descriptive”. The Broken Stick model was used as the null model, with individuals randomly distributed among observed species.

The form of the best fit models was identified for all stands for a subset of “functional” groups where there were at least twenty species collected in each stand to maintain statistical power. The subset of functional groups included saproxylic, trap type and diet (fungivores and predators only). We used a χ^2^ test to compare observed with expected occurrences of best fit models for each functional group. We used a nominal logistic regression on JMP [42] to determine if stand age affected the likelihood of a particular model occurring.

Although the Zipf-Mandelbrot and Zipf models are commonly the best fit to rank abundance data, their parameters lack robustness [43,44]. Instead, we used Mouillot and Lepretre’s [45] *p*_1_ and *p*_2_ to obtain indices that behave similarly to the Zipf-Mandelbrot β and γ. β and γ showed highly erratic patterns and are non-independent, so *p*_1_ and *p*_2_ are recommended as alternatives [45]. Here, *p*_1_ describes the evenness of an assemblage, -*p*_2_ is correlated with γ or ecosystem predictability, i.e., the average probability of the appearance of a species [41]. *p*_1_/*p*_2_ is negatively correlated with β, which represents niche diversity, i.e., the diversity of the environment [45]. *p*_1_ was examined only through the analysis of *p*_1_/*p*_2_ as *p*_1_ is known to correlate with Pielou’s J [45].

We used ANCOVA on JMP [42] to determine the effect of the predictors functional group and stand age (log-transformed) on the response variables species richness, abundance, Shannon’s diversity index (Shannon’s H) and Pielou’s evenness index (Pielou’s J) (calculated on vegan in the R program), predictability (*p*_2_) and niche diversity (*p*_1_/*p*_2_). To compare these response variables amongst trap types, for which we had data only for three of the five stand ages, we used a two-way ANOVA on JMP, with the predictors stand age and trap type. In particular, we were interested in whether there were interactions between functional group/trap type and age that suggested that the importance of stand age differed amongst groups. All data met the assumptions of homogeneity of variances before or after log transformation.

Because reserves were larger than younger stands, there was a risk that differences in species richness might result from the species-area effect, so we tested the effect of stand area on species richness.

## Results

We collected 42 457 individuals belonging to 656 species in the flight intercept traps and 9451 individuals from 279 species in the emergence traps. Total species richness of the functional groups in flight intercept traps (used for most analyses) was: cambium consumers, 42 species; detritivores, 24; fungivores, 167; predators, 125; non-saproxylic species, 217; facultative saproxylics, 159; obligate saproxylics, 271; red-listed, 42 (50% fungivores); and non-red-listed (“common”), 614 species.

### Rank abundance models

Descriptive models proved a better fit to the data than niche-apportionment models. The Zipf-Mandelbrot and Zipf models were the best models (lowest AIC) for all groups examined, although the Dominance Pre-emption model was highest ranked for one of forty-five stands for the emergence trap data. Chi-square tests showed that the Zipf-Mandelbrot model was the best model significantly more often than expected for most functional groups (Table 2). The exception to this was the emergence trap data, where the Zipf model was the best fit in 74% of cases.

**Table 2:** Summary of model fits for common functional groups, showing: the percentage of best fit models across the forty-five age classes for each type of model; best fit model; the χ^2^ test of whether the frequency of the model that fit best most often differed from expected; and the test of whether the best model changed with age.

	**% Best Fit (BF) Model**					**BF**	**BF Frequency**		**Change in BF with age**	
**Functional group**	**Niche**		**Descriptive**							
	**BS**	**DP**	**LN**	**Zipf**	**ZM**		**χ^2^_(1)_**	***p***	**χ^2^_(1)_**	***p***
***Saproxylic***										
Non-saproxylic	0	0	0	31	69	ZM	67.2	**<0.001**	2.1	0.147
Facultative	0	0	0	40	60	ZM	45.0	**<0.001**	0.6	0.429
Obligatory	0	0	0	11	89	ZM	133.5	**<0.001**	0.6	0.440
***Diet***										
Fungivore	0	0	0	18	82	ZM	108.9	**<0.001**	5.2	**0.022**
Predator	0	0	0	9	91	ZM	142.2	**<0.001**	2.3	0.317
***Trap***										
Emergence	0	4	0	74	22	Zipf	49.3	**<0.001**	8.5	0.076
Flight-intercept	0	0	0	7	93	ZM	88.9	**<0.001**	4.9	0.086

Models are: niche-apportionment models: BS = Broken stick; DP = Dominance pre-emption; and descriptive models: LN = Lognormal; Zipf = Zipf; ZM = Zipf-Mandelbrot. BF = best fit.

The occurrence of the Zipf model as best model increased with age for fungivores (Table 2), with the model being most prevalent for mature production stands (56% of mature stands). The opposite pattern was observed for beetles collected in emergence traps, with the Zipf-Mandelbrot model increasing in occurrence with stand age, although not significantly. No trends were evident for any other groups (Table 2).

### Traditional diversity indices

**Figure 1:**
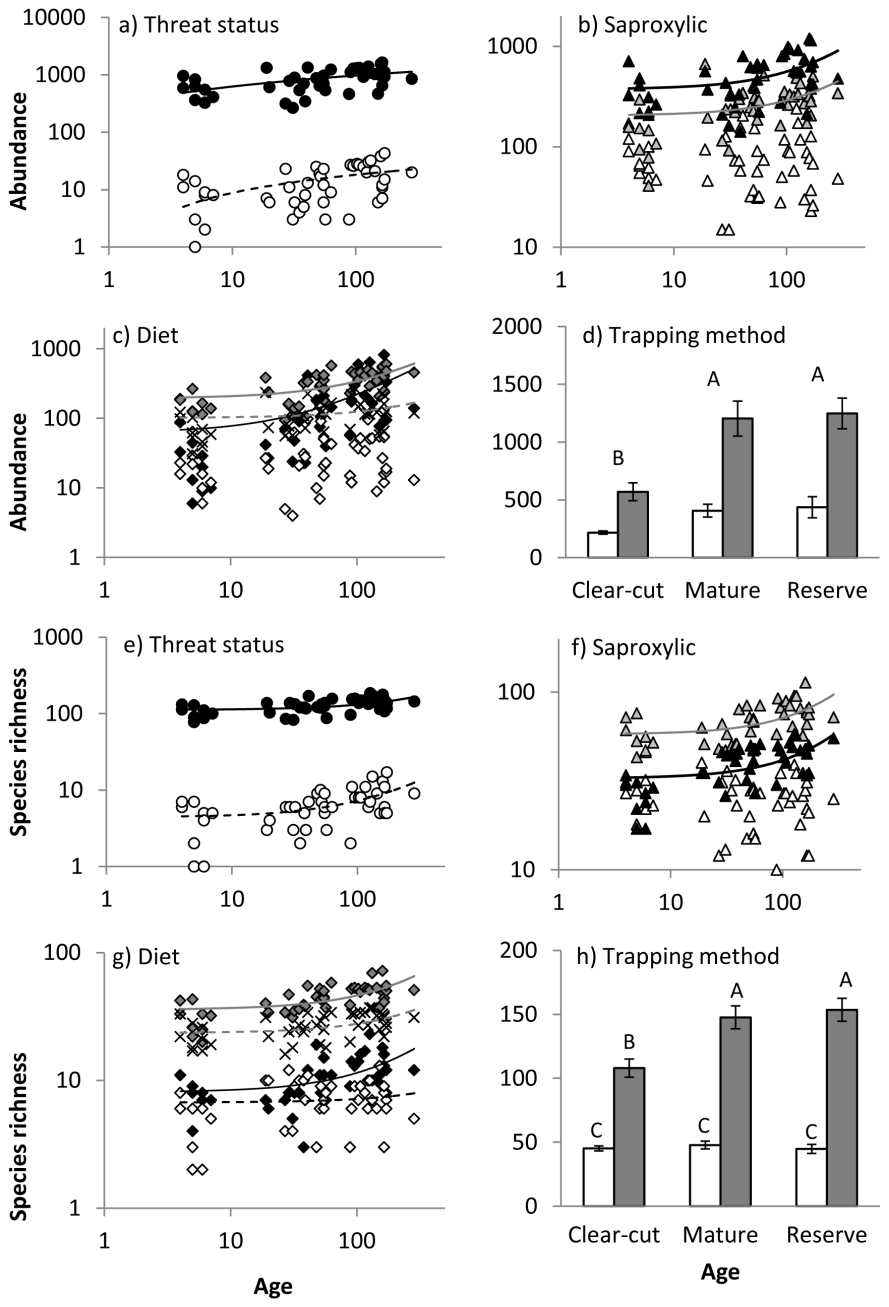
Relationships between stand age and abundance and stand age and species richness for beetle functional groups based on : a) and e) threat status; b) and f) degree to which species are dependent on dead wood; c) and g) diet; and d) and h) trapping method. Symbols are as follows: threat status: common species (●); red-listed species (○); ***saproxylic***: non-saproxylic (Δ); facultative saproxylic (▲); obligatory saproxylic (▲); diet: cambium consumers (♦); detritivores (◊); fungivores (♦); predators (×); trap type: emergence = white bars; flight intercept = grey bars. Only significant or near-significant regression lines are shown. Lines may appear curved due to the log scale of the y-axis.

**Figure 2:**
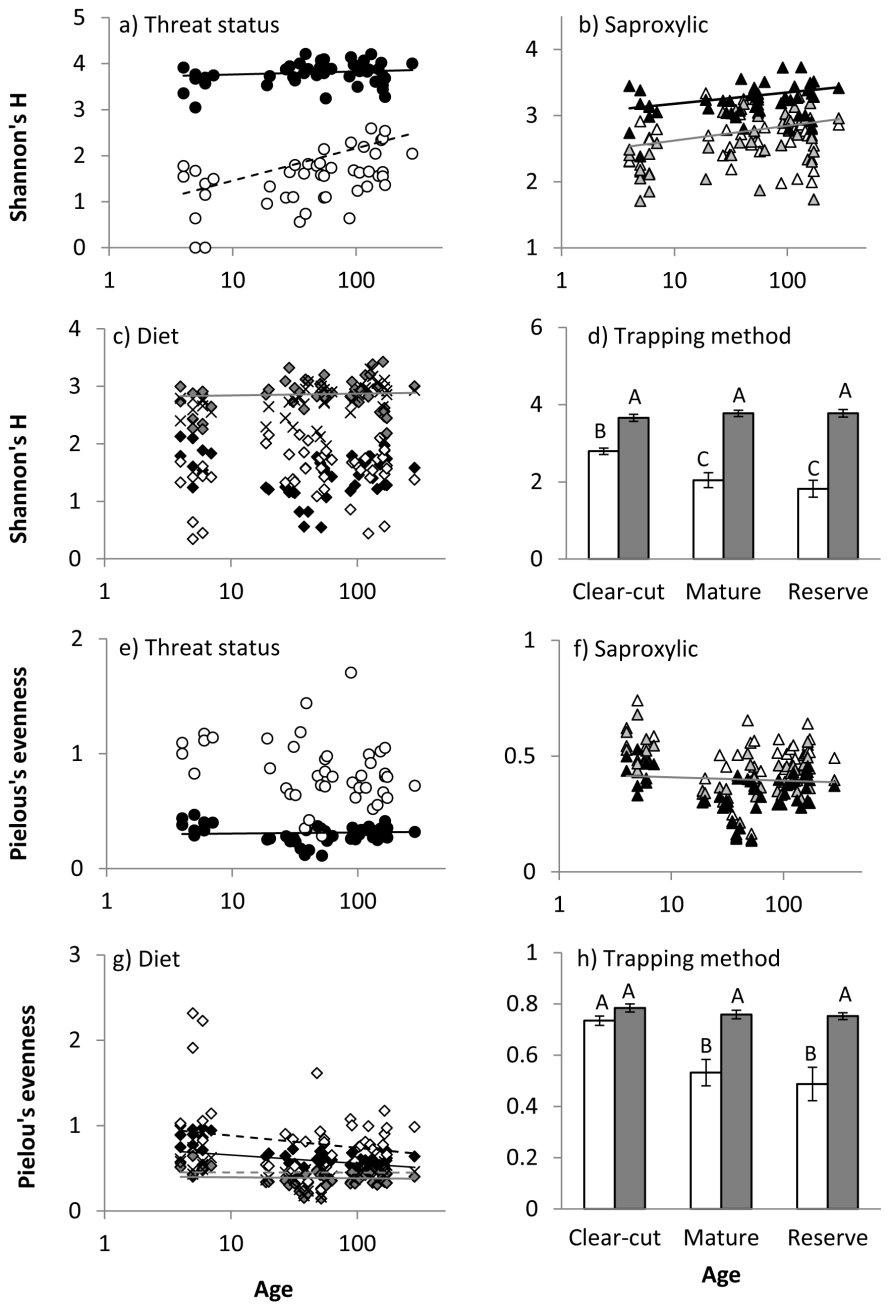
Relationships between stand age and Shannon’s H and stand age and Pielou’s evenness index for beetle functional groups based on: a) and e) threat status; b) and f) degree to which species are dependent on dead wood; c) and g) diet; and d) and h) trapping method. Symbols as for Figure 1. Only significant or near-significant regression lines are shown. Lines may appear curved due to the log scale of the y-axis.

Our test of the relationship between species richness and area for all species showed that area did not significantly predict species richness (R^2^ = 0.06; F_(1,43)_ = 2.65; p = 0.110) and the larger size of reserve sites was therefore unlikely to have influenced our results. Both stand age and functional group significantly affected traditional diversity measures for most sets of functional groups and interactions between functional group and age were common (Table 3, Figures 1 and 2). Most functional group sets increased in species richness, abundance and Shannon’s H with age, although not always significantly so. Pielou’s evenness tended to decrease with stand age. Variance explained by R^2^ ranged from weak (0.11) to moderate (0.50), indicating that unmeasured variables are also likely to be important.

**Table 3:** Analysis of the effect of functional group (FG), stand age and their interaction on measures of diversity.

	***Threatstatus***			***Saproxylic***			***Diet***			***Traptype***	
	**Dev.**	***p***		**Dev.**	***p***		**Dev.**	***p***		**Dev.**	***p***
**Abundance**											
FG	1053.3	**<0.001**		250.0	**<0.001**		233.5	**<0.001**		36.8	**<0.001**
Age	17.5	**<0.001**		22.3	**<0.001**		57.8	**<0.001**		85.8	**<0.001**
Age*FG	1.6	0.213		6.7	**0.036**		27.9	**<0.001**		0.2	0.900
**Sp. richness**											
FG	2574.8	**<0.001**		383.2	**<0.001**		1342.5	**<0.001**		23.7	**<0.001**
Age	22.8	**<0.001**		25.9	**<0.001**		54.0	**<0.001**		465.7	**<0.001**
Age*FG	5.8	**0.016**		11.6	**0.003**		5.6	0.130		8.8	**0.012**
	**F**	***p***		**F**	***p***		**F**	***p***		**F**	***p***
**Shannon’s H**											
FG	717.7	**<0.001**		42.5	**<0.001**		206.6	**<0.001**		179.5	**<0.001**
Age	18.4	**<0.001**		13.9	**<0.001**		4.9	**0.028**		5.1	**0.010**
FG*Age	7.5	**0.008**		2.1	0.128		1.4	0.241		8.6	**0.001**
**Evenness**											
FG	99.8	**<0.001**		14.9	**<0.001**		41.5	**<0.001**		37.1	**<0.001**
Age	10.1	**0.002**		5.6	**0.020**		20.4	**<0.001**		8.4	**0.001**
FG*Age	7.2	**0.009**		0.9	0.395		3.3	**0.023**		5.0	**0.011**
**p_1_/p_2_**											
FG				10.49	**<0.001**		189.9	**<0.001**		1.4	0.248
Age				9.36	**0.003**		20.83	**<0.001**		2.8	0.071
FG*Age				1.35	0.264		3.81	**0.011**		2.4	0.101
**p_2_**											
FG				34.61	**<0.001**		66.77	**<0.001**		0.0	0.933
Age				0.09	0.759		1.32	0.251		6.2	**0.004**
FG*Age				1.27	0.284		2.32	0.077		5.0	**0.011**

All response variables except Pielou’s evenness, Shannon’s H were log-transformed prior to analysis.

Significant interactions between stand age and functional group, which indicated that species with different ecological roles had different age-diversity slopes, were evident for a range of diversity measures and functional groups (Table 3). Interactions for the threat status group indicated differences in the slopes or significance of relationships with stand age between common and red-listed species (Table 4, Figures 1a,e, 2a,e). For saproxylic groups, the interaction indicated that non-saproxylic species responded differently from facultative and obligatory saproxylic species: no traditional metrics (i.e., abundance, species richness, Shannon’s H and Pielou’s evenness) changed significantly with age for non-saproxylic species (Table 4, Figures 1b,f, 2b,f). The evenness of obligatory saproxylic species also did not change with stand age. For diet groups, a significant interaction indicated that detritivore abundance and Shannon’s H did not respond to stand age, while that of other groups did (Table 4, Figures 1c,g, 2c,g). Amongst diet groups, Shannon’s H increased with age only for predators.

**Table 4:** R^2^ and F values, best fit lines and significance of relationship between response variables and log(age) in regression analyses.

	**R^2^**	**F**		**Best fit**		**R^2^**	**F**		**Best fit**		**R^2^**	**F**		**Best fit**
**Response:**	***Abundance***					***Speciesrichness***					***Shannon’sH***			
Common species	0.27	15.7	***	y=348.4x + 276.7		0.28	17.1	***	y=26.2x + 83.4		0.07	3.2	†	y=0.12x + 3.57
Red-listed species	0.24	13.6	***	y=9.4x -0.6		0.27	15.6	***	y=3.3x + 1.1		0.26	15.2	***	y=0.56x + 0.60
Non-saproxylic	0.00	0.0				0.00	0.1				0.02	1.0		
Facultative saproxylic	0.27	16.0	***	y=123.5x + 66.3		0.32	20.3	***	y=16.9x + 40.4		0.18	9.6	**	y=0.35x + 2.07
Obligatory saproxylic	0.22	12.2	**	y=231.8x + 133.7		0.46	37.2	***	y=13.3x + 17.6		0.10	5.0	*	y=0.16x + 2.93
Cambium consumers	0.33	21.4	***	y=204.9x -154.9		0.28	16.8	***	y=4.1x + 3.9		0.01	0.4		
Detritivores	0.03	1.2				0.07	3.4	†	y=1.3x + 4.9		0.05	2.2		
Fungivores	0.46	36.6	***	y=183.9x + 3.0		0.50	42.8	***	y=14.5x + 19.8		0.06	2.6		
Predators	0.17	8.5	**	y=42.6x + 49.1		0.24	13.4	***	y=5.4x + 17.9		0.11	5.4	*	y=0.19x + 2.42
**Response:**	***Pielou’sEvenness***					***P*_*1*_*/P*_*2*_**					***P*_*2*_**			
Common species	0.04	1.8				0.01	0.4				0.18	9.6		
Red-listed species	0.17	8.2	**	y=-0.34x + 1.47									**	y=-0.09x -0.11
Non-saproxylic	0.00	0.1				0.15	6.6	*	y=-10.2x + 29.4		0.02	0.9		
Facultative saproxylic	0.11	5.5	*	y=-0.07x + 0.52		0.02	0.8				0.25	14.0		
Obligatory saproxylic	0.06	2.7				0.00	0.1				0.11	5.4	***	y=-0.37x -0.15
Cambium consumers	0.31	19.3	***	y=-0.16x + 0.90									*	y=-0.12x -0.20
Detritivores	0.14	6.9	*	y=-0.30x + 1.36										
Fungivores	0.10	5.0	*	y=-0.06x + 0.50		0.11	5.4	*	y=21.4x -5.8		0.17	8.7		
Predators	0.06	2.9	†	y=-0.05x + 0.54		0.02	0.9				0.07	3.5	**	y=-0.30x -0.25

*** *p* < 0.001, **p < 0.01, *p < 0.05, † 0.1<*p*<0.05. For the line of best fit, x = log(age), y = response.

ANOVAs revealed interactions between trap type and age for most traditional diversity measures (Table 3). Species richness and abundance were particularly high in flight intercept traps in mature production stands and old-growth reserves, compared with clear-cuts, but relatively constant across stand ages for emergence traps (Figures 1d, h). Shannon’s H and Pielou’s evenness were low in mature production stands and old-growth reserves, relative to clear-cuts, but only for species collected in emergence traps (Figures 2d, h).

### Model-based indices

**Figure 3:**
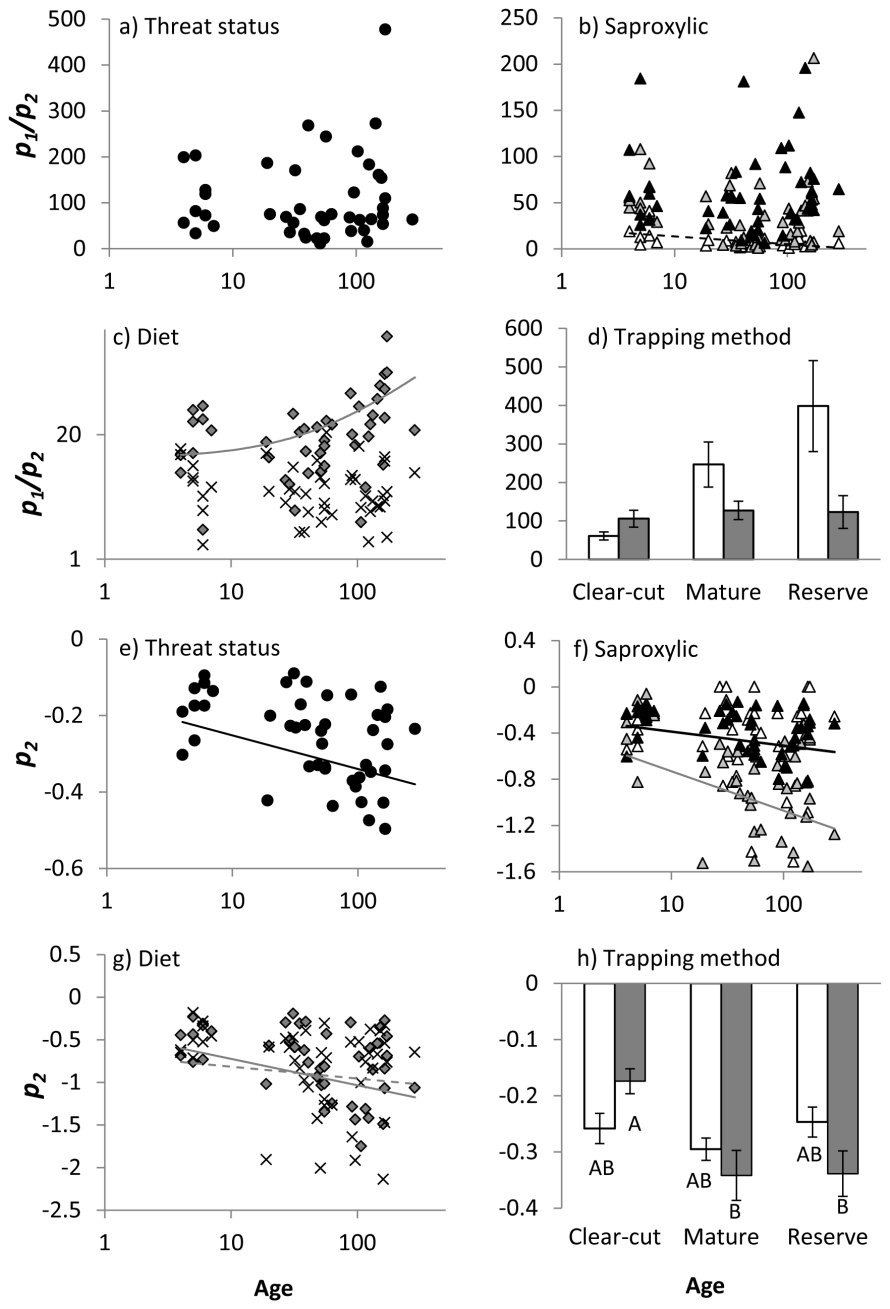
Relationships between stand age and Mouillot and Lepretre’s (1999) derived parameters for the Zipf-Mandelbrot distribution for beetle functional groups based on: a) and e) threat status; b) and f) degree to which species are dependent on dead wood; c) and g) diet; and d) and h) trapping method. Functional groups where several age classes supported less than twenty species (red-listed species, detritivores and cambium consumers) were excluded. Symbols as for Figure 1. Only significant or near-significant regression lines are shown. Lines may appear curved due to the log scale of the y-axis.

Model-based indices suggested patterns that were largely consistent with traditional indices, but gave some further insights into community structure (Tables 3 & 4, Figure 3). To reiterate, *p*_2_ is correlated with ecosystem predictability and *p*_1_/*p*_2_ is negatively correlated with niche diversity. The effect of threat status on the response of model-based diversity indices to age could not be tested as red-listed species were too rare to create valid models for individual sites. For common species, index *p*_2_ decreased with stand age, indicating increasing predictability, while there was no relationship between stand age and *p*_1_/*p*_2_ (Figures 3a,e). Amongst saproxylic groups, the relationship between stand age and *p*_1_/*p*_2_ was significant only for non-saproxylic species, which declined with stand age, although the relationship was relatively weak (R^2^ = 0.15, Figure 3b). The slope of the *p*_2_ – stand age relationship was lowest and the relationship strongest for facultative species, suggesting that their assemblages were most predictable, while non-saproxylic species showed no relationship (Figure 3f).

For diet, *p*_1_/*p*_2_ increased with stand age for fungivores, but R^2^ was low and the relationship was not significant for other taxa (Figure 3c). This suggested an increase in niche diversity with age for fungivores. Interactions between age and functional group resulted from a stronger relationship between *p*_2_ and stand age for fungivores than for predators (Table 4, Figure 3g). This indicated increasing predictability of fungivore assemblages with age. For trap type, *p*_1_/*p*_2_ showed a non-significant trend to increase with stand age for emergence traps, suggesting an increase in niche diversity (Figure 3d). Interactions between trap type and stand age were significant or close to significant for *p*_2_ (Figure 3h). *p*_2_ was lowest in clear-cut stands for flight intercept traps, indicating high predictability relative to other stand ages.

## Discussion

Boreal beetle assemblages responded strongly to forest succession. The best-fit rank abundance curve did not suggest a strong role for biotic interactions in structuring assemblages, i.e., statistical models provided a better fit than niche-based models for almost all functional groups and stand ages. The slopes of several traditional and derived diversity measures depended on functional group. In particular, species dependent on resources that are slow to accumulate, e.g., saproxylic and fungivorous species, showed the steepest slopes. Succession thus affected the diversity of different functional groups differently, which is an important consideration in conservation decision-making, where the aim is commonly to maximise diversity across landscapes (e.g. [46]).

### Rank-abundance models and succession

Of the rank-abundance models tested, the Zipf-Mandelbrot model was most commonly the best fit model, as for previous studies (e.g., [47]). Best model differed only for trap type, with the Zipf model the most common best fit for emergence traps, while the Zipf-Mandelbrot model was most common for flight intercept data. The Zipf model is a special case of the Zipf-Mandelbrot model, where β=0 [45]. β is thought to represent niche diversity, so the prevalence of the Zipf model for species caught in emergence traps suggests that niche diversity may be less important in determining the shape of the rank abundance curve for emergence trap species. This seems plausible as the experimental logs on which emergence traps were placed were relatively uniform, i.e., of the same decomposition stage and tree species, thus supporting fewer niches than the area trapped by the less targeted flight intercept traps.

Rank abundance fits did not suggest that biotic interactions were important in structuring beetle assemblages: the dominance pre-emption and broken stick models, which describe competitively structured assemblages, were very rarely amongst the top models. The Zipf-Mandelbrot and Zipf models are descriptive models [40], and their superior fit cannot be attributed to biotic interactions such as niche partitioning. However, this is not necessarily indicative of a neutral mechanism of assemblage structuring as previous studies suggest that multiple mechanisms can lead to the same model [19,48–52].

The best-fit model changed with stand age for fungivores, with the Zipf model becoming more prevalent than the Zipf-Mandelbrot model in older stands. This indicates that niche diversity (β, represented by p_1_/p_2_) may have become less important with increasing stand age for this group. Previous studies suggest that the abundance and diversity of fungi increases with the availability of dead wood [53–55], which depends on stand age [56,57], thus providing a greater diversity of habitats, so this is a surprising outcome. Closer inspection of the data revealed a non-significant trend for the Zipf model to appear in mature production stands. In contrast to other stand types, mature production stands have previously been selectively logged and are therefore likely to support a reduced diversity of dead wood habitats [57,58]. Previous studies using experimental logs suggest that the saproxylic beetle fauna in these sites is similar to that in old-growth forests [5,12,59,60]. However, patterns detected here suggest that assemblage structure may differ subtly for fungivores, with dominance being greater in mature production stands.

Although different mechanisms can lead to the same model [19,52], the overall consistency in model fit across successional stages (with the exception of fungivores) suggests there is unlikely to be any fundamental change in the mechanisms structuring assemblages of most functional groups over time. It also suggests that the structure of different functional groups may result from similar mechanisms. Previous studies have revealed a variety of responses of rank-abundance models to succession, ranging from no change [47,61], change in fit with succession [62] to a change through three model types with age [63,64]. The consistency in model fits shown here suggests that there is no increase in competitive dominance through succession for beetles in boreal forests (cf. intermediate disturbance hypothesis [21,22]). This is in contrast to patterns for ants in this system [65], and may be a result of the high dispersal ability and habitat specificity of beetles. In addition, short summers and low temperatures at high latitudes (63-65^°^ N in this study) could limit the ability of beetles to exploit all available resources [59,66–68].

### Successional trends in diversity indices amongst functional groups

Most indicators suggested that diversity increased with succession. Traditional measures, including abundance, species richness and Shannon’s diversity index increased with stand age for most functional groups, while Pielou’s evenness index decreased for some groups. Niche diversity (*p*_1_/*p*_2_) increased over time for many taxa, particularly those dependent on dead wood substrates and fungi. The absolute magnitude of ecosystem predictability (-*p*_2_, i.e., the average probability of the appearance of a species) also increased with time. Although this might be expected to increase opportunities for species to encounter one-another and therefore compete, there was no indication of niche-apportionment models increasing in prevalence as the best-fit model.

Interactions between functional group and stand age were common, reflecting differences in diversity-succession slopes amongst functional groups. Threat status affected the slope of the relationship for all diversity measures, with red-listed species consistently showing shallower slopes than common species for abundance and species richness. The Shannon’s H of red-listed species responded more strongly to stand age than did that of common species, suggesting that stands in later successional stages support more diverse assemblages of red-listed species (see also 12). Although the evenness of red-listed species declined with stand age, evenness of common species remained constant. The selection of red-listed species may be biased to some extent toward those species we believe are at risk, resulting in listing of more species dependent on late successional forest stages, which are increasingly rare in the landscape [69,70]. However, given that even common species increased in abundance and diversity with stand age, it is logical to assume that older stands support a significant number of threatened species. Taking into account the well-known relationship between area and species richness [10], the greater species richness in older stands is likely to have resulted from the greater area occupied by older stands in landscapes of the recent past [8,9,71], and the high diversity of substrates accumulating in older stands [72]. The loss of previously dominant mature age classes from the landscape and the mismatch with current species richness suggests that extinction debts [73,74] may still be owed in this landscape.

Differences in responses between saproxylic and non-saproxylic beetle species suggest that the loss of older age classes affects a specific set of species. While obligate and facultatively saproxylic beetles increased in species richness, abundance and Shannon’s H with age, these measures remained relatively constant for non-saproxylic species. The volume and diversity of dead wood substrates increases with stand age [12,56,57,75], thus increasing niche availability for species dependent on that resource. The failure of non-saproxylic species to respond positively to increases in stand age suggests that, although there may be turnover in the types of non-woody resources available, this does not result in any increase in overall niche availability. Conservation targeting only non-saproxylic beetle species would thus result in landscapes with a relatively even distribution of age classes, contrasting strongly with the older stand dominated landscape required for the conservation of saproxylic species.

Patterns for diet-based functional groups reflected those for saproxylic functional groups: diversity measures for cambium consumers and fungivores, which are most closely tied to dead wood substrates, responded strongly to stand age. In contrast, detritivores and predators showed weaker responses, possibly reflecting the broader range of resources available to them across the succession. Cambium consumers are clearly dead wood dependent, so their response to stand age is as predicted. Fungus diversity and abundance increases with stand age, in parallel with the diversity and volume of dead wood [58,76,77]. Many fungivorous beetles have high diet specificity [78–80], so an increase in their diversity may reflect the increase in diversity of their food resources. Mouillot and Lepretre’s [45] indices suggest that, for fungivores, niche diversity and ecosystem predictability increase with stand age. The volume of detritus also accumulates with stand age, up to a point [81]. In contrast, the diversity of plants, including deciduous trees, decreases over time in boreal forests [82], presumably resulting in a lower diversity of leaf litter. This trade-off between the volume and diversity of litter may be responsible for the failure of detritivore diversity to respond to stand age.

We did not survey prey species, so it is difficult to determine whether the lack of relationship between stand age and predatory beetles results from prey abundance or another limiting factor. Predatory beetles show mixed diversity responses to successional stage, with decreases [83–85], U-shaped relationships [86] and increases over time [5,60] all observed. Most predators are generalised to some extent in their use of prey (p. 279 [87]), so it is reasonable to assume that prey abundance is similar to total abundance and that composition of prey species is less important than the total biomass. Insects develop more rapidly in the warmer temperatures found in open habitats such as clear-cuts and younger stands [88], so resource availability may trade-off with succession such that predator populations remain relatively consistent.

The abundance and species richness of beetles collected in emergence traps was much lower than that of beetles collected in flight intercept traps. Emergence traps were placed on spruce logs of relatively uniform size, harvested in the same logging operation. Emerging beetles thus presented a limited subset of beetle diversity. Stands of all ages support a greater diversity of dead wood substrates than was targeted by our emergence traps (see 12,57), so the difference in abundance is not surprising. Although abundance increased with stand age for both emerging and flying beetles, species richness increased only for flying beetles. Evenness and Shannon’s H decreased with stand age for emerging beetles, but not flying beetles, suggesting that assemblage dominance increased for this group. Dead wood diversity and volume is higher in older forests than clear-cuts [12,57] and the diversity of flying saproxylic beetles would have tracked the diversity of this resource. Differences in dead wood availability between mature production stands and old-growth stands are also distinct, but were not mirrored in captures of flying or emerging insects. This could be because many of the species collected in flight intercepts are ‘tourists’, which are passing through the habitat, in search of suitable substrates on which to feed and reproduce. Protected mature forests and old-growth forests are superficially similar, with tall trees and closed canopies. Beetles attracted to superficial habitat cues may be attracted to mature production forests, even if critical resources are rare, so such forests could act as ecological traps [89–91]. However, beetle attraction to suboptimal sites suggests clear pathways for biodiversity conservation through restoration of the diversity of substrates [5,92]. Given the large coverage of mature production forests relative to old-growth forests, substrate restoration [5,60,92,93] could improve landscape-scale conservation of old-growth species.

## Conclusions

This study shows clear differences in responses to forest succession between beetle species, depending on their ecological roles. This suggests there will be differential susceptibility to rotation periods among functional groups. The preference of most functional groups for older successional stages suggests that the historically larger area of old-growth forests has promoted a greater richness of species dependent on this habitat, as would be predicted from the species-area effect, whether this is due to greater habitat heterogeneity or area *per se*. Greater species richness may also have resulted from a greater diversity of niches per unit area in older stands, where a greater variety of dead wood substrates accumulate. Identification of the specific drivers of diversity for specific functional groups will aid in their conservation in managed landscapes. In some cases, these are known and management actions are already in place in some systems (e.g. [93]). Although responses for saproxylic and fungivorous beetle species were as predicted, other taxa, such as predators and detritivores, may display more complex responses to stand age and it is important that we also identify the key habitat variables to which they respond. Differences between trapping methods suggest that the faunal component sampled is critical to our interpretation of species responses and that care should be taken in considering the generality of findings from studies limited to single trapping methods.

## Supporting Information

Appendix S1 **Functional group allocation of species collected in flight-intercept and emergence traps.** x = high degree of certainty; o = lower degree of certainty in allocation to functional groups.
(DOCX)
