# Supplementary material for: Functional Roles Affect Diversity-Succession Relationships for Boreal Beetles
Source: PLoS One. 2013 Aug 20;8(8):e72764. doi: 10.1371/journal.pone.0072764 (PMC3748087; doi:10.1371/journal.pone.0072764)
Supplement: Appendix S1 — Functional group allocation of species collected in flight-intercept and emergence traps. x = high degree of certainty; o = lower degree of certainty in allocation to functional groups. (DOCX) [file pone.0072764.s001.docx]

**Appendix I:** Functional group allocation of species collected in flight-intercept and emergence traps. x = high degree of certainty; o = lower degree of certainty in allocation to functional groups.

| **Species** | **Cambium consumer** | **Detritivore** | **Fungivore** | **Herbivore** | **Predator** | **Sapwood consumer** | **Red-listed** | **Common** | **Obligatory saproxylic** | **Facultative saproxylic** | **Non-saproxylic** | **Flight-intercept trap** | **Emergence traps** |
| --- | --- | --- | --- | --- | --- | --- | --- | --- | --- | --- | --- | --- | --- |
| *Acidota crenata* |  |  |  |  | x |  |  | x |  | x |  | 15 | 2 |
| *Acrulia inflata* |  |  | o |  |  |  |  | x |  | x |  | 48 | 25 |
| *Agathidium nigripenne* |  |  | x |  |  |  |  | x |  | x |  | 5 | 0 |
| *Agathidium rotundatum* |  |  | x |  |  |  |  | x |  | x |  | 61 | 13 |
| *Agathidium seminulum* |  |  | x |  |  |  |  | x |  | x |  | 658 | 35 |
| *Aleochara stichai* |  |  |  |  | x |  |  | x |  | x |  | 4 | 0 |
| *Amphicyllus globus* |  |  | x |  |  |  |  | x |  | x |  | 2 | 0 |
| *Anthophagus caraboides* |  |  |  |  | o |  |  | x |  | x |  | 3 | 3 |
| *Anthrenus scrophulariae* |  | x |  |  |  |  |  | x |  | x |  | 1 | 0 |
| *Arpidiphorus orbiculatus* |  |  | x |  |  |  |  | x |  | x |  | 154 | 19 |
| *Atheta sg Alaobia gagatina* |  | o | o |  | x |  |  | x |  | x |  | 25 | 0 |
| *Atheta sg Alaobia sodalis* |  | o | x |  | x |  |  | x |  | x |  | 23 | 3 |
| *Atheta sg Alaobia sparreschneideri* |  | o | o |  | x |  |  | x |  | x |  | 2 | 0 |
| *Atheta sg Atheta acutangula* |  | o | o |  | x |  |  | x |  | x |  | 1 | 4 |
| *Atheta sg Atheta brunneipennis* |  | o | o |  | x |  |  | x |  | x |  | 2 | 1 |
| *Atheta sg Atheta coriaria* |  | o | o |  | x |  |  | x |  | x |  | 1 | 0 |
| *Atheta sg Atheta crassicornis* |  | o | o |  | x |  |  | x |  | x |  | 25 | 1 |
| *Atheta sg Atheta euryptera* |  | o | o |  | x |  |  | x |  | x |  | 35 | 11 |
| *Atheta sg Atheta harwoodi* |  | o | o |  | x |  |  | x |  | x |  | 2 | 2 |
| *Atheta sg Atheta hypnorum* |  | o | x |  | x |  |  | x |  | x |  | 18 | 5 |
| *Atheta sg Atheta incognita* |  | o | o |  | x |  |  | x |  | x |  | 37 | 2 |
| *Atheta sg Atheta nigricornis* |  | o | o |  | x |  |  | x |  | x |  | 113 | 10 |
| *Atheta sg Atheta paracrassicornis* |  | o | o |  | x |  |  | x |  | x |  | 5 | 3 |
| *Atheta sg Atheta pilicornis* |  | o | o |  | x |  |  | x |  | x |  | 19 | 9 |
| *Atheta sg Atheta procera* |  | o | o |  | x |  |  | x |  | x |  | 15 | 0 |
| *Atheta sg Bessobia excellens* |  | o | o |  | x |  |  | x |  | x |  | 1 | 0 |
| *Atheta sg Dimetrota cinnamoptera* |  | o | o |  | x |  |  | x |  | x |  | 2 | 0 |
| *Atheta sg Dimetrota nigripes* |  | o | o |  | x |  |  | x |  | x |  | 1 | 0 |
| *Atheta sg Microdota benickiella* |  | o | o |  | x |  |  | x |  | x |  | 1 | 0 |
| *Atheta sg Microdota subtilis* |  | o | o |  | x |  |  | x |  | x |  | 62 | 1 |
| *Atheta sg Traumoecia picipes* |  | o | o |  | x |  |  | x |  | x |  | 19 | 0 |
| *Atheta sg Xenota myrmecobia* |  | o | o |  | x |  |  | x |  | x |  | 286 | 3 |
| *Athous subfuscus* |  |  |  |  | x |  |  | x |  | x |  | 287 | 7 |
| *Atomaria apicalis* |  |  | x |  |  |  |  | x |  | x |  | 5 | 0 |
| *Atomaria atrata* |  |  | x |  |  |  |  | x |  | x |  | 8 | 4 |
| *Atomaria fuscata* |  |  | x |  |  |  |  | x |  | x |  | 13 | 0 |
| *Atomaria morio* |  |  | x |  |  |  |  | x |  | x |  | 1 | 0 |
| *Atomaria ornata* |  |  | x |  |  |  |  | x |  | x |  | 411 | 0 |
| *Atomaria peltata* |  |  | x |  |  |  |  | x |  | x |  | 8 | 2 |
| *Atomaria pulchra* |  |  | x |  |  |  |  | x |  | x |  | 43 | 22 |
| *Atomaria pusilla* |  |  | x |  |  |  |  | x |  | x |  | 1 | 0 |
| *Atomaria turgida* |  |  | x |  |  |  |  | x |  | x |  | 2 | 0 |
| *Autalia impressa* |  |  | o |  |  |  |  | x |  | x |  | 30 | 1 |
| *Bisnius puella* |  |  |  |  | o |  |  | x |  | x |  | 31 | 0 |
| *Bolitochara pulchra* |  |  | x |  |  |  |  | x |  | x |  | 19 | 20 |
| *Bryoporus cernuus* |  |  |  |  | o |  |  | x |  | x |  | 13 | 0 |
| *Bryoporus crassicornis* |  |  |  |  | o |  |  | x |  | x |  | 2 | 1 |
| *Bryoporus punctipennis* |  |  |  |  | o |  |  | x |  | x |  | 16 | 1 |
| *Caenoscelis ferruginea* |  |  | x |  |  |  |  | x |  | x |  | 1 | 0 |
| *Corticaria abietorum* |  |  | x |  |  |  |  | x |  | x |  | 4 | 10 |
| *Corticaria ferruginea* |  |  | x |  |  |  |  | x |  | x |  | 4 | 0 |
| *Corticaria rubripes* |  |  | x |  |  |  |  | x |  | x |  | 133 | 11 |
| *Corticarina fuscula* |  |  | x |  |  |  |  | x |  | x |  | 5 | 4 |
| *Corticarina obfuscata* |  |  | x |  |  |  |  | x |  | x |  | 11 | 2 |
| *Cortinicara gibbosa* |  |  | x |  |  |  |  | x |  | x |  | 9 | 2 |
| *Coryphium angusticolle* |  | x |  |  |  |  |  | x |  | x |  | 19 | 3 |
| *Cryptophagus abietis* |  |  | x |  |  |  |  | x |  | x |  | 122 | 9 |
| *Cryptophagus badius* |  |  | x |  |  |  |  | x |  | x |  | 4 | 0 |
| *Cryptophagus dentatus* |  |  | x |  |  |  |  | x |  | x |  | 87 | 6 |
| *Cryptophagus dorsalis* |  |  | x |  |  |  |  | x |  | x |  | 7 | 0 |
| *Cryptophagus lapponicus* |  |  | x |  |  |  |  | x |  | x |  | 2301 | 2 |
| *Cryptophagus longitarsis* |  |  | x |  |  |  |  | x |  | x |  | 18 | 5 |
| *Cryptophagus pilosus* |  |  | x |  |  |  |  | x |  | x |  | 1 | 0 |
| *Cryptophagus saginatus* |  |  | x |  |  |  |  | x |  | x |  | 2 | 0 |
| *Cryptophagus scanicus* |  |  | x |  |  |  |  | x |  | x |  | 72 | 4 |
| *Cryptophagus setulosus* |  |  | x |  |  |  |  | x |  | x |  | 2 | 3 |
| *Cryptophagus subdepressus* |  |  | x |  |  |  |  | x |  | x |  | 19 | 0 |
| *Cryptophagus tuberculosus* |  |  | x |  |  |  |  | x |  | x |  | 6 | 0 |
| *Cychramus variegatus* |  |  | x |  |  |  |  | x |  | x |  | 1 | 0 |
| *Deliphrum tectum* |  |  |  |  | o |  |  | x |  | x |  | 48 | 0 |
| *Dendrophilus pygmaeus* |  |  |  |  | x |  |  | x |  | x |  | 13 | 1 |
| *Dromius agilis* |  |  |  |  | x |  |  | x |  | x |  | 27 | 0 |
| *Dromius fenestratus* |  |  |  |  | x |  |  | x |  | x |  | 3 | 0 |
| *Enicmus fungicola* |  |  | x |  |  |  |  | x |  | x |  | 433 | 1 |
| *Epuraea aestiva* |  |  | x |  |  |  |  | x |  | x |  | 66 | 0 |
| *Epuraea binotata* |  |  | x |  |  |  |  | x |  | x |  | 12 | 0 |
| *Epuraea melina* |  | x | x |  |  |  |  | x |  | x |  | 5 | 0 |
| *Epuraea placida* |  | x | x |  |  |  |  | x |  | x |  | 2 | 0 |
| *Epuraea rufomarginata* |  |  | x |  |  |  |  | x |  | x |  | 3 | 0 |
| *Epuraea terminalis* |  |  | x |  |  |  |  | x |  | x |  | 1 | 0 |
| *Epuraea unicolor* |  | x | x |  |  |  |  | x |  | x |  | 1 | 0 |
| *Euconnus claviger* |  |  |  |  | x |  |  | x |  | x |  | 4 | 0 |
| *Euplectus karsteni* |  |  |  |  | x |  |  | x |  | x |  | 1 | 1 |
| *Euplectus nanus* |  |  |  |  | x |  |  | x |  | x |  | 3 | 3 |
| *Euplectus piceus* |  |  |  |  | x |  |  | x |  | x |  | 7 | 1 |
| *Gabrius expectatus* |  |  |  |  | x |  |  | x |  | x |  | 660 | 37 |
| *Glischrochilus hortensis* |  | x | x |  |  |  |  | x |  | x |  | 4 | 0 |
| *Gnathoncus buyssoni* |  |  |  |  | x |  |  | x |  | x |  | 13 | 0 |
| *Gnathoncus nannetensis* |  |  |  |  | x |  |  | x |  | x |  | 3 | 0 |
| *Gyrophaena affinis* |  |  | x |  |  |  |  | x |  | x |  | 26 | 0 |
| *Gyrophaena fasciata* |  |  | x |  |  |  |  | x |  | x |  | 6 | 0 |
| *Haploglossa marginalis* |  |  | o |  |  |  |  | x |  | x |  | 6 | 0 |
| *Haploglossa villosula* |  |  | o |  |  |  |  | x |  | x |  | 414 | 0 |
| *Holobus flavicornis* |  |  |  |  | x |  |  | x |  | x |  | 12 | 3 |
| *Ischnoglossa elegantula* |  |  | o |  |  |  |  | x |  | x |  | 185 | 382 |
| *Latridius consimilis* |  |  | x |  |  |  |  | x |  | x |  | 9 | 4 |
| *Latridius nidicola* |  |  | x |  |  |  |  | x |  | x |  | 3 | 1 |
| *Liogluta microptera* |  |  | o |  |  |  |  | x |  | x |  | 31 | 2 |
| *Liotrichus affinis* |  |  |  |  |  |  |  | x |  | x |  | 210 | 0 |
| *Lordithon lunulatus* |  |  |  |  | x |  |  | x |  | x |  | 68 | 8 |
| *Lordithon thoracicus* |  |  |  |  | x |  |  | x |  | x |  | 35 | 1 |
| *Lordithon trinotatus* |  |  |  |  | x |  |  | x |  | x |  | 27 | 0 |
| *Megasternum obscurum* |  |  |  |  | x |  |  | x |  | x |  | 96 | 0 |
| *Megatoma undata* |  |  |  |  | x |  |  | x |  | x |  | 4 | 0 |
| *Mycetoporus punctus* |  |  |  |  | x |  |  | x |  | x |  | 13 | 0 |
| *Mycetoporus rufescens* |  |  |  |  | x |  |  | x |  | x |  | 3 | 1 |
| *Nevraphes coronatus* |  |  |  |  | x |  |  | x |  | x |  | 2 | 7 |
| *Omalium caesum* |  |  |  |  | o |  |  | x |  | x |  | 2 | 2 |
| *Omalium rivulare* |  |  |  |  | o |  |  | x |  | x |  | 1 | 0 |
| *Omalium strigicolle* |  |  |  |  | o |  |  | x |  | x |  | 1 | 0 |
| *Orthoperus atomus* |  |  | x |  |  |  |  | x |  | x |  | 52 | 2 |
| *Orthoperus punctatus* |  |  | x |  |  |  |  | x |  | x |  | 5 | 3 |
| *Oxypoda alternans* |  |  | o |  |  |  |  | x |  | x |  | 25 | 12 |
| *Oxypoda annularis* |  |  | o |  |  |  |  | x |  | x |  | 1 | 0 |
| *Oxypoda hansseni* |  |  | o |  |  |  |  | x |  | x |  | 13 | 0 |
| *Oxypoda skalitzkyi* |  |  | o |  |  |  |  | x |  | x |  | 10 | 0 |
| *Oxypoda soror* |  |  | o |  |  |  |  | x |  | x |  | 5 | 4 |
| *Oxypoda spectabilis* |  |  | o |  |  |  |  | x |  | x |  | 3 | 0 |
| *Philonthus addendus* |  |  |  |  | o |  |  | x |  | x |  | 1 | 0 |
| *Philonthus decorus* |  |  |  |  | o |  |  | x |  | x |  | 4 | 0 |
| *Philonthus marginatus* |  |  |  |  | o |  |  | x |  | x |  | 1 | 0 |
| *Phyllodrepa nigra* |  |  | o |  |  |  |  | x |  | x |  | 2 | 0 |
| *Pocadius ferrugineus* |  |  | x |  |  |  |  | x |  | x |  | 14 | 0 |
| *Potosia cuprea ssp metallica* |  | x |  |  |  |  |  | x |  | x |  | 129 | 0 |
| *Proteinus brachypterus* |  |  |  |  | o |  |  | x |  | x |  | 43 | 0 |
| *Ptenidium formicetorum* |  |  | x |  |  |  |  | x |  | x |  | 1 | 0 |
| *Pterostichus strenuus* |  |  |  |  | x |  |  | x |  | x |  | 2 | 0 |
| *Ptinus villiger* | o |  |  |  |  |  |  | x |  | x |  | 1 | 0 |
| *Quedius brevis* |  |  |  |  | x |  |  | x |  | x |  | 58 | 2 |
| *Quedius cruentus* |  |  |  |  | o |  |  | x |  | x |  | 8 | 0 |
| *Quedius fuliginosus* |  |  |  |  | o |  |  | x |  | x |  | 1 | 3 |
| *Quedius lucidulus* |  |  |  |  | o |  |  | x |  | x |  | 13 | 0 |
| *Quedius mesomelinus* |  |  |  |  | o |  |  | x |  | x |  | 6 | 1 |
| *Quedius tenellus* |  |  |  |  | o |  |  | x |  | x |  | 416 | 3 |
| *Quedius xanthopus* |  |  |  |  | o |  |  | x |  | x |  | 14 | 8 |
| *Rhizophagus dispar* |  |  |  |  | x |  |  | x |  | x |  | 61 | 124 |
| *Scaphisoma agaricinum* |  |  | o |  |  |  |  | x |  | x |  | 2151 | 455 |
| *Selatosomus aeneus* |  |  |  | x |  |  |  | x |  | x |  | 84 | 0 |
| *Selatosomus impressus* |  |  |  |  | x |  |  | x |  | x |  | 33 | 0 |
| *Sepedophilus constans* |  |  | x |  |  |  |  | x |  | x |  | 1 | 0 |
| *Sepedophilus immaculatus* |  |  | x |  |  |  |  | x |  | x |  | 3 | 9 |
| *Sepedophilus littoreus* |  |  | x |  |  |  |  | x |  | x |  | 626 | 78 |
| *Sepedophilus marshami* |  |  | x |  |  |  |  | x |  | x |  | 13 | 0 |
| *Sepedophilus testaceus* |  |  | x |  |  |  |  | x |  | x |  | 5 | 4 |
| *Sphaerites glabratus* |  |  |  |  | o |  |  | x |  | x |  | 6 | 0 |
| *Sphindus dubius* |  |  | x |  |  |  |  | x |  | x |  | 59 | 2 |
| *Stenichnus bicolor* |  |  |  |  | x |  |  | x |  | x |  | 145 | 72 |
| *Stephostethus pandellei* |  |  | x |  |  |  |  | x |  | x |  | 1 | 0 |
| *Stephostethus rugicollis* |  |  | x |  |  |  |  | x |  | x |  | 44 | 0 |
| *Syntomium aeneum* |  |  |  |  | o |  |  | x |  | x |  | 20 | 0 |
| *Tachinus subterraneus* |  |  | o |  | o |  |  | x |  | x |  | 4 | 0 |
| *Trichophya pilicornis* |  |  | o |  |  |  |  | x |  | x |  | 3 | 0 |
| *Tyrus mucronatus* |  |  | o |  |  |  |  | x |  | x |  | 5 | 11 |
| *Agathidium discoideum* |  |  | x |  |  |  | x |  |  | x |  | 13 | 0 |
| *Agathidium mandibulare* |  |  | x |  |  |  | x |  |  | x |  | 42 | 4 |
| *Agathidium nigrinum* |  |  | x |  |  |  | x |  |  | x |  | 5 | 3 |
| *Agathidium pallidum* |  |  | x |  |  |  | x |  |  | x |  | 35 | 2 |
| *Corticaria interstitialis* |  |  | x |  |  |  | x |  |  | x |  | 27 | 10 |
| *Acrostiba borealis* |  |  |  |  | o |  |  | x |  |  | x | 18 | 0 |
| *Acrotona pygmaea* |  |  |  |  | o |  |  | x |  |  | x | 1 | 0 |
| *Adalia conglomerata* |  |  |  |  | x |  |  | x |  |  | x | 1 | 0 |
| *Adalia decempunctata* |  |  |  |  | x |  |  | x |  |  | x | 1 | 0 |
| *Agabus bipustulatus* |  |  |  |  | x |  |  | x |  |  | x | 1 | 0 |
| *Aleochara albovillosa* |  | x |  |  | x |  |  | x |  |  | x | 5 | 0 |
| *Aleochara brevipennis* |  |  |  |  | x |  |  | x |  |  | x | 2 | 0 |
| *Aleochara fumata* |  |  |  |  | x |  |  | x |  |  | x | 7 | 0 |
| *Aleochara moerens* |  |  |  |  | x |  |  | x |  |  | x | 41 | 0 |
| *Aloconota gregaria* |  |  |  |  | o |  |  | x |  |  | x | 1 | 0 |
| *Altica longicollis* |  |  |  | x |  |  |  | x |  |  | x | 1 | 0 |
| *Amara communis* |  |  |  | x | x |  |  | x |  |  | x | 1 | 0 |
| *Amara lunicollis* |  |  |  | x | x |  |  | x |  |  | x | 3 | 0 |
| *Amischa analis* |  |  |  |  | x |  |  | x |  |  | x | 138 | 0 |
| *Amischa bifoveolata* |  |  |  |  | o |  |  | x |  |  | x | 1 | 0 |
| *Anatis ocellata* |  |  |  |  | x |  |  | x |  |  | x | 1 | 0 |
| *Anopleta depressicollis* |  |  |  |  |  |  |  | x |  |  | x | 2 | 0 |
| *Anotylus clavatus* |  |  |  |  | o |  |  | x |  |  | x | 2 | 0 |
| *Anotylus nitidulus* |  |  |  |  | o |  |  | x |  |  | x | 1 | 0 |
| *Anotylus rugosus* |  |  |  |  | o |  |  | x |  |  | x | 2 | 0 |
| *Antherophagus pallens* |  |  |  | x |  |  |  | x |  |  | x | 2 | 0 |
| *Anthobium atrocephalum* |  |  |  |  | o |  |  | x |  |  | x | 6 | 0 |
| *Anthophagus alpinus* |  |  |  |  | x |  |  | x |  |  | x | 1 | 0 |
| *Anthophagus omalinus* |  |  |  |  | x |  |  | x |  |  | x | 106 | 0 |
| *Aphodius ater* |  | x |  |  |  |  |  | x |  |  | x | 5 | 0 |
| *Aphodius borealis* |  | x |  |  |  |  |  | x |  |  | x | 69 | 0 |
| *Aphodius depressus* |  | x |  |  |  |  |  | x |  |  | x | 62 | 0 |
| *Aphodius fimetarius* |  | x |  |  |  |  |  | x |  |  | x | 1 | 0 |
| *Aphodius lapponum* |  | x |  |  |  |  |  | x |  |  | x | 28 | 0 |
| *Aphodius nemoralis* |  | x |  |  |  |  |  | x |  |  | x | 72 | 0 |
| *Aphodius pusillus* |  | x |  |  |  |  |  | x |  |  | x | 50 | 0 |
| *Aphodius rufipes* |  | x |  |  |  |  |  | x |  |  | x | 382 | 0 |
| *Apion seniculus* |  |  |  | x |  |  |  | x |  |  | x | 4 | 0 |
| *Aploderus caelatus* |  |  |  |  | o |  |  | x |  |  | x | 1 | 0 |
| *Atheta sg Atheta diversa* |  | o | o |  | x |  |  | x |  |  | x | 1 | 0 |
| *Atheta sg Atheta divisa* |  | o | o |  | x |  |  | x |  |  | x | 1 | 0 |
| *Atheta sg Atheta laevicauda* |  | o | o |  | x |  |  | x |  |  | x | 5 | 0 |
| *Atheta sg Boreophila eremita* |  | o | o |  | x |  |  | x |  |  | x | 177 | 0 |
| *Atheta sg Dimetrota aeneipennis* |  | o | o |  | x |  |  | x |  |  | x | 32 | 0 |
| *Atheta sg Lypoglossa lateralis* |  | o | o |  | x |  |  | x |  |  | x | 32 | 0 |
| *Atheta sg Microdota excelsa* |  | o | o |  | x |  |  | x |  |  | x | 2 | 0 |
| *Atheta sg Microdota spatuloides* |  | o | o |  | x |  |  | x |  |  | x | 1 | 0 |
| *Atheta sg Mocyta fungi* |  | o | o |  | x |  |  | x |  |  | x | 6 | 0 |
| *Atheta sg Notothecta flavipes* |  | o | o |  | x |  |  | x |  |  | x | 23 | 0 |
| *Atomaria atricapilla* |  |  | x |  |  |  |  | x |  |  | x | 1 | 0 |
| *Atomaria rubella* |  |  | x |  |  |  |  | x |  |  | x | 6 | 0 |
| *Bembidion grapii* |  |  |  |  | x |  |  | x |  |  | x | 1 | 0 |
| *Bembidion lampros* |  |  |  |  | x |  |  | x |  |  | x | 1 | 0 |
| *Bolitobius castaneus* |  |  |  |  | o |  |  | x |  |  | x | 4 | 0 |
| *Bolitobius cingulatus* |  |  |  |  | o |  |  | x |  |  | x | 3 | 0 |
| *Bradycellus caucasicus* |  |  |  |  | o |  |  | x |  |  | x | 1 | 0 |
| *Bryaxis bulbifer* |  |  |  |  | x |  |  | x |  |  | x | 11 | 0 |
| *Byrrhus fasciatus* |  |  |  | x |  |  |  | x |  |  | x | 1 | 0 |
| *Byturus tomentosus* |  |  |  | x |  |  |  | x |  |  | x | 4 | 0 |
| *Calathus micropterus* |  |  |  |  | x |  |  | x |  |  | x | 6 | 0 |
| *Calodera aethiops* |  |  | o |  |  |  |  | x |  |  | x | 5 | 0 |
| *Cantharis obscura* |  |  |  | x | x |  |  | x |  |  | x | 1 | 0 |
| *Cantharis paludosa* |  |  |  | x | x |  |  | x |  |  | x | 3 | 0 |
| *Carpelimus gracilis* |  | o |  |  |  |  |  | x |  |  | x | 1 | 0 |
| *Catops alpinus* |  | x |  |  |  |  |  | x |  |  | x | 67 | 0 |
| *Catops coracinus* |  | x |  |  |  |  |  | x |  |  | x | 13 | 0 |
| *Catops luteipes* |  | x |  |  |  |  |  | x |  |  | x | 1 | 0 |
| *Catops morio* |  | x |  |  |  |  |  | x |  |  | x | 2 | 0 |
| *Catops nigrita* |  | x |  |  |  |  |  | x |  |  | x | 71 | 0 |
| *Catops tristis* |  | x |  |  |  |  |  | x |  |  | x | 8 | 0 |
| *Cercyon borealis* |  |  |  |  | x |  |  | x |  |  | x | 10 | 0 |
| *Cercyon depressus* |  |  |  |  | x |  |  | x |  |  | x | 1 | 0 |
| *Cercyon impressus* |  |  |  |  | x |  |  | x |  |  | x | 43 | 0 |
| *Cercyon lateralis* |  |  |  |  | x |  |  | x |  |  | x | 8 | 0 |
| *Cercyon pygmaeus* |  |  |  |  | x |  |  | x |  |  | x | 1 | 0 |
| *Cercyon unipunctatus* |  |  |  |  | x |  |  | x |  |  | x | 1 | 0 |
| *Cimberis attelaboides* |  |  |  | x |  |  |  | x |  |  | x | 2 | 0 |
| *Coccinella magnifica* |  |  |  |  | x |  |  | x |  |  | x | 1 | 0 |
| *Coccinella septempunctata* |  |  |  |  | x |  |  | x |  |  | x | 2 | 0 |
| *Coccinula quattuordecimpustulata* |  |  |  |  | x |  |  | x |  |  | x | 3 | 0 |
| *Coeliodes rubicundus* |  |  |  | x |  |  |  | x |  |  | x | 1 | 0 |
| *Corticaria pubescens* |  |  | x |  |  |  |  | x |  |  | x | 1 | 0 |
| *Cryptocephalus labiatus* |  |  |  | x |  |  |  | x |  |  | x | 7 | 0 |
| *Cryptocephalus pini* |  |  |  | x |  |  |  | x |  |  | x | 1 | 0 |
| *Cryptocephalus quadripustulatus* |  |  |  | x |  |  |  | x |  |  | x | 1 | 0 |
| *Cryptophagus confertus* |  |  | x |  |  |  |  | x |  |  | x | 5 | 0 |
| *Cryptopleurum subtile* |  | x |  | x | x |  |  | x |  |  | x | 1 | 0 |
| *Cyphon padi* |  | x |  |  | x |  |  | x |  |  | x | 2 | 0 |
| *Cyphon pubescens* |  | x |  |  | x |  |  | x |  |  | x | 1 | 0 |
| *Cyphon punctipennis* |  | x |  |  | x |  |  | x |  |  | x | 2 | 0 |
| *Cyphon variabilis* |  | x |  |  | x |  |  | x |  |  | x | 10 | 0 |
| *Cytilus sericeus* |  |  |  | x |  |  |  | x |  |  | x | 2 | 0 |
| *Dalopius marginatus* |  | o |  |  |  |  |  | x |  |  | x | 5 | 0 |
| *Deporaus betulae* |  |  |  | x |  |  |  | x |  |  | x | 2 | 0 |
| *Dromius schneideri* |  |  |  |  | x |  |  | x |  |  | x | 2 | 0 |
| *Eanus costalis* |  |  |  |  |  |  |  | x |  |  | x | 26 | 0 |
| *Enochrus affinis* |  |  |  |  | x |  |  | x |  |  | x | 2 | 0 |
| *Erichsonius cinerascens* |  |  |  |  | o |  |  | x |  |  | x | 1 | 0 |
| *Euaesthetus ruficapillus* |  |  |  |  | o |  |  | x |  |  | x | 2 | 0 |
| *Eucnecosum brachypterum* |  |  |  |  | x |  |  | x |  |  | x | 1 | 0 |
| *Euryptilium saxonicum* |  |  | x |  |  |  |  | x |  |  | x | 3 | 0 |
| *Gabrius appendiculatus* |  |  |  |  | o |  |  | x |  |  | x | 4 | 0 |
| *Gabrius trossulus* |  |  |  |  | o |  |  | x |  |  | x | 3 | 0 |
| *Geotrupes stercorarius* |  | x |  |  |  |  |  | x |  |  | x | 2 | 0 |
| *Geotrupes stercorosus* |  | x |  |  |  |  |  | x |  |  | x | 9 | 0 |
| *Harpalus latus* |  |  |  | x |  |  |  | x |  |  | x | 1 | 0 |
| *Helophorus flavipes* |  |  |  |  | x |  |  | x |  |  | x | 1 | 0 |
| *Heterhelus scutellaris* |  |  |  | x |  |  |  | x |  |  | x | 1 | 0 |
| *Hydnobius spinipes* |  |  | x |  |  |  |  | x |  |  | x | 1 | 0 |
| *Hydroporus morio* |  |  |  |  | x |  |  | x |  |  | x | 1 | 0 |
| *Ischnopoda leucopus* |  |  |  |  | o |  |  | x |  |  | x | 1 | 0 |
| *Ischnosoma splendidum* |  |  |  |  | x |  |  | x |  |  | x | 3 | 0 |
| *Lathrobium elongatum* |  |  |  |  | o |  |  | x |  |  | x | 4 | 0 |
| *Lathrobium fulvipenne* |  |  |  |  | o |  |  | x |  |  | x | 3 | 0 |
| *Lathrobium rufipenne* |  |  |  |  | o |  |  | x |  |  | x | 3 | 0 |
| *Leiodes inordinata* |  |  | x |  |  |  |  | x |  |  | x | 3 | 0 |
| *Leiodes lucens* |  |  | x |  |  |  |  | x |  |  | x | 7 | 0 |
| *Leiodes obesa* |  |  | x |  |  |  |  | x |  |  | x | 1 | 0 |
| *Leiodes polita* |  |  | x |  |  |  |  | x |  |  | x | 1 | 0 |
| *Leiodes puncticollis* |  |  | x |  |  |  |  | x |  |  | x | 3 | 0 |
| *Leiodes punctulata* |  |  | x |  |  |  |  | x |  |  | x | 6 | 0 |
| *Leiodes silesiaca* |  |  | x |  |  |  |  | x |  |  | x | 7 | 0 |
| *Leptacinus formicetorum* |  |  |  |  | o |  |  | x |  |  | x | 4 | 0 |
| *Lesteva longelytrata* |  |  |  |  | o |  |  | x |  |  | x | 3 | 0 |
| *Limnius volckmari* |  |  |  | o |  |  |  | x |  |  | x | 1 | 0 |
| *Limonius aeneoniger* |  |  |  |  |  |  |  | x |  |  | x | 5 | 0 |
| *Liogluta micans* |  |  | o |  |  |  |  | x |  |  | x | 3 | 0 |
| *Liogluta pagana* |  |  | o |  |  |  |  | x |  |  | x | 1 | 0 |
| *Lyprocorrhe anceps* |  |  |  |  | o |  |  | x |  |  | x | 1 | 0 |
| *Lythraria salicariae* |  |  |  | x |  |  |  | x |  |  | x | 2 | 0 |
| *Megarthrus depressus* |  |  |  |  | o |  |  | x |  |  | x | 5 | 0 |
| *Megarthrus fennicus* |  |  |  |  | o |  |  | x |  |  | x | 2 | 0 |
| *Megarthrus nigrinus* |  |  |  |  | o |  |  | x |  |  | x | 1 | 0 |
| *Megarthrus nitidulus* |  |  |  |  | o |  |  | x |  |  | x | 3 | 0 |
| *Megarthrus sinuatocollis* |  |  |  |  | o |  |  | x |  |  | x | 16 | 0 |
| *Meotica exilis* |  |  | o |  |  |  |  | x |  |  | x | 2 | 0 |
| *Microcara testacea* |  |  |  | o |  |  |  | x |  |  | x | 1 | 0 |
| *Monotoma conicicollis* |  |  |  |  | x |  |  | x |  |  | x | 4 | 0 |
| *Mycetoporus lepidus* |  |  |  |  | x |  |  | x |  |  | x | 132 | 0 |
| *Myllaena intermedia* |  |  |  |  | o |  |  | x |  |  | x | 9 | 0 |
| *Myrmetes paykulli* |  |  |  |  | x |  |  | x |  |  | x | 11 | 0 |
| *Myzia oblongoguttata* |  |  |  |  | x |  |  | x |  |  | x | 7 | 0 |
| *Nephus bisignatus* |  |  |  |  | x |  |  | x |  |  | x | 4 | 0 |
| *Nicrophorus vespilloides* |  | x |  |  | x |  |  | x |  |  | x | 238 | 0 |
| *Notiophilus biguttatus* |  |  |  |  | x |  |  | x |  |  | x | 2 | 0 |
| *Ochthephilum fracticorne* |  |  |  |  | o |  |  | x |  |  | x | 1 | 0 |
| *Oiceoptoma thoracica* |  | o |  |  | x |  |  | x |  |  | x | 4 | 0 |
| *Olophrum boreale* |  |  |  |  | o |  |  | x |  |  | x | 3 | 0 |
| *Olophrum consimile* |  |  |  |  | o |  |  | x |  |  | x | 2 | 0 |
| *Olophrum rotundicolle* |  |  |  |  | o |  |  | x |  |  | x | 1 | 0 |
| *Omalium septentrionis* |  |  |  |  | o |  |  | x |  |  | x | 5 | 0 |
| *Omosita depressa* |  | x | x |  |  |  |  | x |  |  | x | 4 | 0 |
| *Orithales serraticornis* |  |  |  |  |  |  |  | x |  |  | x | 16 | 0 |
| *Otiorhynchus nodosus* |  |  |  | x |  |  |  | x |  |  | x | 2 | 0 |
| *Otiorhynchus scaber* |  |  |  | x |  |  |  | x |  |  | x | 5 | 0 |
| *Oxypoda acuminata* |  |  | o |  |  |  |  | x |  |  | x | 1 | 0 |
| *Oxypoda brevicornis* |  |  | o |  |  |  |  | x |  |  | x | 13 | 0 |
| *Oxypoda exoleta* |  |  | o |  |  |  |  | x |  |  | x | 1 | 0 |
| *Oxypoda longipes* |  |  | o |  |  |  |  | x |  |  | x | 1 | 0 |
| *Oxypoda nigricornis* |  |  | o |  |  |  |  | x |  |  | x | 1 | 0 |
| *Oxypoda opaca* |  |  | o |  |  |  |  | x |  |  | x | 3 | 0 |
| *Oxypoda procerula* |  |  | o |  |  |  |  | x |  |  | x | 3 | 0 |
| *Oxypoda strandi* |  |  | o |  |  |  |  | x |  |  | x | 1 | 0 |
| *Oxytelus laqueatus* |  |  |  |  | o |  |  | x |  |  | x | 3 | 0 |
| *Philhygra arctica* |  |  |  |  | o |  |  | x |  |  | x | 6 | 0 |
| *Philhygra britteni* |  |  |  |  | o |  |  | x |  |  | x | 1 | 0 |
| *Philhygra deformis* |  |  |  |  | o |  |  | x |  |  | x | 3 | 0 |
| *Philhygra palustris* |  |  |  |  | o |  |  | x |  |  | x | 1 | 0 |
| *Philonthus lederi* |  |  |  |  | o |  |  | x |  |  | x | 20 | 0 |
| *Philonthus succicola* |  |  |  |  | o |  |  | x |  |  | x | 15 | 0 |
| *Philonthus varians* |  |  |  |  | o |  |  | x |  |  | x | 1 | 0 |
| *Philorhizus sigma* |  |  |  |  | x |  |  | x |  |  | x | 1 | 0 |
| *Phratora vitellinae* |  |  |  | x |  |  |  | x |  |  | x | 1 | 0 |
| *Platydracus fulvipes* |  |  |  |  | o |  |  | x |  |  | x | 1 | 0 |
| *Platydracus latebricola* |  |  |  |  | x |  |  | x |  |  | x | 1 | 0 |
| *Podabrus alpinus* |  |  |  |  | o |  |  | x |  |  | x | 3 | 0 |
| *Polydrusus pilosus* |  |  |  | x |  |  |  | x |  |  | x | 10 | 0 |
| *Polydrusus ruficornis* |  |  |  | x |  |  |  | x |  |  | x | 5 | 0 |
| *Polydrusus undatus* |  |  |  | x |  |  |  | x |  |  | x | 6 | 0 |
| *Prosternon tessellatum* |  |  |  | x |  |  |  | x |  |  | x | 2 | 0 |
| *Pteroloma forsstroemi* |  |  |  |  | o |  |  | x |  |  | x | 6 | 0 |
| *Quedius boopoides* |  |  |  |  | o |  |  | x |  |  | x | 1 | 0 |
| *Quedius boops* |  |  |  |  | o |  |  | x |  |  | x | 1 | 0 |
| *Quedius cinctus* |  |  |  |  | o |  |  | x |  |  | x | 2 | 0 |
| *Quedius nitipennis* |  |  |  |  | x |  |  | x |  |  | x | 2 | 0 |
| *Rhagonycha atra* |  |  |  |  | x |  |  | x |  |  | x | 16 | 0 |
| *Rhagonycha elongata* |  |  |  |  | x |  |  | x |  |  | x | 7 | 0 |
| *Rhagonycha limbata* |  |  |  |  | x |  |  | x |  |  | x | 6 | 0 |
| *Rhamphus pulicarius* |  |  |  | x |  |  |  | x |  |  | x | 1 | 0 |
| *Rhynchaenus rusci* |  |  |  | x |  |  |  | x |  |  | x | 1 | 0 |
| *Rhynchaenus stigma* |  |  |  | x |  |  |  | x |  |  | x | 1 | 0 |
| *Schistoglossa curtipennis* |  |  | o |  |  |  |  | x |  |  | x | 4 | 0 |
| *Schistoglossa gemina* |  |  | o |  |  |  |  | x |  |  | x | 4 | 0 |
| *Sciodrepoides fumatus* |  | x |  |  |  |  |  | x |  |  | x | 14 | 0 |
| *Sciodrepoides watsoni* |  | x |  |  |  |  |  | x |  |  | x | 308 | 0 |
| *Scymnus abietis* |  |  |  |  | x |  |  | x |  |  | x | 1 | 0 |
| *Scymnus jakowlewi* |  |  |  |  | x |  |  | x |  |  | x | 2 | 0 |
| *Scymnus limbatus* |  |  |  |  | x |  |  | x |  |  | x | 3 | 0 |
| *Scymnus nigrinus* |  |  |  |  | x |  |  | x |  |  | x | 2 | 0 |
| *Selatosomus melancholicus* |  |  |  |  |  |  |  | x |  |  | x | 1 | 0 |
| *Sepedophilus pedicularius* |  |  | x |  |  |  |  | x |  |  | x | 3 | 0 |
| *Sericus brunneus* |  |  |  |  |  |  |  | x |  |  | x | 226 | 0 |
| *Stenus clavicornis* |  |  |  |  | x |  |  | x |  |  | x | 3 | 0 |
| *Syneta betulae* |  |  |  | x |  |  |  | x |  |  | x | 20 | 0 |
| *Tachinus atripes* |  |  | o |  | o |  |  | x |  |  | x | 1 | 0 |
| *Tachinus elongatus* |  |  | o |  | o |  |  | x |  |  | x | 45 | 0 |
| *Tachinus humeralis* |  |  | o |  | o |  |  | x |  |  | x | 1 | 0 |
| *Tachinus laticollis* |  | x |  |  |  |  |  | x |  |  | x | 76 | 0 |
| *Tachinus marginellus* |  |  | x |  |  |  |  | x |  |  | x | 9 | 0 |
| *Tachinus pallipes* |  |  | o |  | o |  |  | x |  |  | x | 327 | 0 |
| *Tachinus proximus* |  |  | o |  | o |  |  | x |  |  | x | 62 | 0 |
| *Tachinus rufipes* |  |  | x |  | x |  |  | x |  |  | x | 9 | 0 |
| *Tachyerges decoratus* |  |  |  | x |  |  |  | x |  |  | x | 2 | 0 |
| *Tachyporus chrysomelinus* |  |  |  |  | o |  |  | x |  |  | x | 3 | 0 |
| *Tachyporus transversalis* |  |  |  |  | o |  |  | x |  |  | x | 5 | 0 |
| *Thalycra fervida* |  |  | x | o |  |  |  | x |  |  | x | 7 | 0 |
| *Trechus rubens* |  |  |  |  | x |  |  | x |  |  | x | 6 | 0 |
| *Trichiusa immigrata* |  |  |  |  |  |  |  | x |  |  | x | 1 | 0 |
| *Zorochros minimus* |  | x |  |  | x |  |  | x |  |  | x | 2 | 0 |
| *Zyras humeralis* |  |  |  |  | x |  |  | x |  |  | x | 1 | 16 |
| *Tachinus elegans* |  |  | o |  | o |  | x |  |  |  | x | 65 | 0 |
| *Abdera affinis* |  |  | x |  |  |  |  | x | x |  |  | 4 | 0 |
| *Abdera flexuosa* |  |  | x |  |  |  |  | x | x |  |  | 6 | 0 |
| *Abdera triguttata* |  |  | x |  |  |  |  | x | x |  |  | 8 | 28 |
| *Absidia schoenherri* |  |  |  |  | x |  |  | x | x |  |  | 285 | 32 |
| *Acmaeops septentrionis* | x |  |  |  |  | x |  | x | x |  |  | 2 | 0 |
| *Agathidium pisanum* |  |  | x |  |  |  |  | x | x |  |  | 5 | 51 |
| *Alosterna tabacicolor* |  |  |  |  |  | x |  | x | x |  |  | 1 | 0 |
| *Ampedus balteatus* |  |  |  |  | x | x |  | x | x |  |  | 90 | 3 |
| *Ampedus nigrinus* |  |  |  |  | x | x |  | x | x |  |  | 1029 | 10 |
| *Ampedus pomorum* |  |  |  |  | x | x |  | x | x |  |  | 2 | 0 |
| *Ampedus tristis* |  |  |  |  | x | x |  | x | x |  |  | 410 | 227 |
| *Anaspis arctica* |  |  |  |  | x |  |  | x | x |  |  | 1 | 0 |
| *Anaspis bohemica* |  |  |  |  | x |  |  | x | x |  |  | 15 | 12 |
| *Anaspis marginicollis* |  |  |  |  | x |  |  | x | x |  |  | 34 | 41 |
| *Anaspis rufilabris* |  |  |  |  | x |  |  | x | x |  |  | 7 | 69 |
| *Anisotoma axillaris* |  |  | x |  |  |  |  | x | x |  |  | 107 | 4 |
| *Anisotoma castanea* |  |  | x |  |  |  |  | x | x |  |  | 145 | 7 |
| *Anisotoma glabra* |  |  | x |  |  |  |  | x | x |  |  | 163 | 3 |
| *Anisotoma humeralis* |  |  | x |  |  |  |  | x | x |  |  | 74 | 3 |
| *Anobium thomsoni* |  |  |  |  |  | x |  | x | x |  |  | 1 | 0 |
| *Anomognathus cuspidatus* |  | x |  |  | x |  |  | x | x |  |  | 17 | 0 |
| *Anoplodera reyi* |  |  |  |  |  | x |  | x | x |  |  | 5 | 0 |
| *Anoplodera sanguinolenta* |  |  |  |  |  | x |  | x | x |  |  | 2 | 0 |
| *Anoplodera virens* |  |  |  |  |  | x |  | x | x |  |  | 1 | 0 |
| *Anthaxia quadripunctata* | x |  |  |  |  |  |  | x | x |  |  | 3 | 2 |
| *Asemum striatum* | x |  |  |  |  | x |  | x | x |  |  | 7 | 1 |
| *Atomaria bella* |  |  | x |  |  |  |  | x | x |  |  | 179 | 58 |
| *Atomaria subangulata* |  |  | x |  |  |  |  | x | x |  |  | 15 | 55 |
| *Atomaria umbrina* |  |  | x |  |  |  |  | x | x |  |  | 21 | 7 |
| *Atrecus affinis* |  |  |  |  | x |  |  | x | x |  |  | 181 | 6 |
| *Atrecus longiceps* |  |  |  |  | x |  |  | x | x |  |  | 9 | 12 |
| *Atrecus pilicornis* |  |  |  |  | x |  |  | x | x |  |  | 108 | 110 |
| *Bibloporus bicolor* |  |  |  |  | x |  |  | x | x |  |  | 373 | 35 |
| *Bolitochara mulsanti* |  |  | o |  |  |  |  | x | x |  |  | 2 | 9 |
| *Bolitophagus reticulatus* |  |  | x |  |  |  |  | x | x |  |  | 2 | 0 |
| *Buprestis rustica* |  |  |  |  |  | x |  | x | x |  |  | 1 | 0 |
| *Calopus serraticornis* |  |  |  |  |  | x |  | x | x |  |  | 6 | 0 |
| *Cardiophorus ruficollis* |  | x |  |  |  | x |  | x | x |  |  | 4 | 0 |
| *Cerylon ferrugineum* |  |  | x |  |  |  |  | x | x |  |  | 318 | 26 |
| *Cerylon histeroides* |  |  | x |  |  |  |  | x | x |  |  | 972 | 126 |
| *Chrysanthia geniculata* |  |  |  |  |  | x |  | x | x |  |  | 1 | 0 |
| *Cis alter* |  |  | x |  |  |  |  | x | x |  |  | 8 | 0 |
| *Cis bidentatus* |  |  | x |  |  |  |  | x | x |  |  | 95 | 1 |
| *Cis boleti* |  |  | x |  |  |  |  | x | x |  |  | 369 | 1 |
| *Cis comptus* |  |  | x |  |  |  |  | x | x |  |  | 70 | 3 |
| *Cis glabratus* |  |  | x |  |  |  |  | x | x |  |  | 6 | 5 |
| *Cis hanseni* |  |  | x |  |  |  |  | x | x |  |  | 1 | 0 |
| *Cis hispidus* |  |  | x |  |  |  |  | x | x |  |  | 155 | 8 |
| *Cis lineatocribratus* |  |  | x |  |  |  |  | x | x |  |  | 18 | 11 |
| *Cis punctulatus* |  |  | x |  |  |  |  | x | x |  |  | 53 | 55 |
| *Corticaria lapponica* |  |  | x |  |  |  |  | x | x |  |  | 5 | 0 |
| *Corticaria lateritia* |  |  | x |  |  |  |  | x | x |  |  | 7 | 2 |
| *Corticaria obsoleta* |  |  | x |  |  |  |  | x | x |  |  | 1 | 0 |
| *Corticaria orbicollis* |  |  | x |  |  |  |  | x | x |  |  | 14 | 7 |
| *Corticeus linearis* |  |  |  |  | x |  |  | x | x |  |  | 2 | 0 |
| *Cryphalus saltuarius* | x |  |  |  |  |  |  | x | x |  |  | 18 | 0 |
| *Cryptolestes abietis* |  |  |  |  | x |  |  | x | x |  |  | 19 | 0 |
| *Cryptolestes alternans* |  |  |  |  | x |  |  | x | x |  |  | 8 | 0 |
| *Cryptophagus confusus* |  |  | x |  |  |  |  | x | x |  |  | 7 | 0 |
| *Cryptophagus plagiatus* |  |  | x |  |  |  |  | x | x |  |  | 1 | 0 |
| *Crypturgus cinereus* | x |  |  |  |  |  |  | x | x |  |  | 6 | 32 |
| *Crypturgus hispidulus* | x |  |  |  |  |  |  | x | x |  |  | 1 | 0 |
| *Crypturgus pusillus* | x |  |  |  |  |  |  | x | x |  |  | 1152 | 7553 |
| *Crypturgus subcribrosus* | x |  |  |  |  |  |  | x | x |  |  | 50 | 3 |
| *Curtimorda maculosa* |  |  | x |  |  |  |  | x | x |  |  | 84 | 230 |
| *Cybocephalus politus* |  |  |  |  | x |  |  | x | x |  |  | 1 | 0 |
| *Dacne bipustulata* |  |  | x |  |  |  |  | x | x |  |  | 390 | 11 |
| *Dadobia immersa* |  |  | o |  |  |  |  | x | x |  |  | 39 | 33 |
| *Dasytes niger* |  |  |  |  | x |  |  | x | x |  |  | 44 | 35 |
| *Dasytes obscurus* |  |  |  |  | o |  |  | x | x |  |  | 14 | 0 |
| *Dendroctonus micans* | x |  |  |  |  |  |  | x | x |  |  | 13 | 1 |
| *Dendrophagus crenatus* |  |  | x |  |  |  |  | x | x |  |  | 144 | 7 |
| *Denticollis linearis* |  |  |  |  | x | x |  | x | x |  |  | 64 | 4 |
| *Dictyoptera aurora* |  |  |  |  | x |  |  | x | x |  |  | 205 | 0 |
| *Dinaraea aequata* |  |  | o |  |  |  |  | x | x |  |  | 6 | 11 |
| *Dinaraea arcana* |  |  | o |  |  |  |  | x | x |  |  | 7 | 12 |
| *Dinaraea linearis* |  |  | o |  |  |  |  | x | x |  |  | 142 | 14 |
| *Dorcatoma dresdensis* |  |  | x |  |  |  |  | x | x |  |  | 15 | 1 |
| *Dryocoetes alni* | x |  |  |  |  |  |  | x | x |  |  | 1 | 0 |
| *Dryocoetes autographus* | x |  |  |  |  |  |  | x | x |  |  | 3278 | 581 |
| *Dryocoetes hectographus* | x |  |  |  |  |  |  | x | x |  |  | 8 | 0 |
| *Endomychus coccineus* |  |  | x |  |  |  |  | x | x |  |  | 3 | 1 |
| *Enicmus rugosus* |  |  | x |  |  |  |  | x | x |  |  | 2126 | 31 |
| *Ennearthron cornutum* |  |  | x |  |  |  |  | x | x |  |  | 19 | 0 |
| *Episernus angulicollis* |  |  |  |  |  | x |  | x | x |  |  | 5 | 1 |
| *Epuraea angustula* |  |  | x |  | x |  |  | x | x |  |  | 30 | 0 |
| *Epuraea biguttata* |  |  | x |  |  |  |  | x | x |  |  | 30 | 0 |
| *Epuraea boreella* |  |  | x |  | x |  |  | x | x |  |  | 15 | 0 |
| *Epuraea deubeli* |  |  | x |  | x |  |  | x | x |  |  | 39 | 0 |
| *Epuraea laeviuscula* |  |  |  |  | x |  |  | x | x |  |  | 5 | 0 |
| *Epuraea longipennis* |  | x | x |  |  |  |  | x | x |  |  | 18 | 0 |
| *Epuraea marseuli* |  | x | x |  |  |  |  | x | x |  |  | 16 | 1 |
| *Epuraea opalizans* |  | x | x |  |  |  |  | x | x |  |  | 2 | 0 |
| *Epuraea pygmaea* |  | x | x |  |  |  |  | x | x |  |  | 189 | 4 |
| *Epuraea silacea* |  |  | x |  |  |  |  | x | x |  |  | 150 | 0 |
| *Epuraea variegata* |  |  | x |  |  |  |  | x | x |  |  | 6 | 33 |
| *Ernobius explanatus* |  |  |  |  |  | x |  | x | x |  |  | 7 | 1 |
| *Euconnus pragensis* |  |  |  |  | x |  |  | x | x |  |  | 5 | 0 |
| *Euglenes pygmaeus* |  |  | x |  |  |  |  | x | x |  |  | 1 | 5 |
| *Euplectus decipiens* |  |  |  |  | x |  |  | x | x |  |  | 6 | 0 |
| *Euplectus punctatus* |  |  |  |  | x |  |  | x | x |  |  | 686 | 61 |
| *Glischrochilus quadripunctatus* |  |  | x |  | x |  |  | x | x |  |  | 14 | 1 |
| *Globicornis emarginata* |  | x |  |  |  |  |  | x | x |  |  | 40 | 1 |
| *Gyrophaena angustata* |  |  | x |  |  |  |  | x | x |  |  | 1 | 0 |
| *Gyrophaena boleti* |  |  | x |  |  |  |  | x | x |  |  | 31 | 0 |
| *Gyrophaena strictula* |  |  | x |  |  |  |  | x | x |  |  | 10 | 41 |
| *Hadreule elongatula* |  |  | x |  |  |  |  | x | x |  |  | 3 | 1 |
| *Hadrobregmus pertinax* |  |  |  |  |  | x |  | x | x |  |  | 8 | 2 |
| *Hallomenus binotatus* |  |  | x |  |  |  |  | x | x |  |  | 100 | 12 |
| *Homalota plana* |  |  | o |  |  |  |  | x | x |  |  | 2 | 0 |
| *Hylastes brunneus* | x |  |  |  |  |  |  | x | x |  |  | 42 | 1 |
| *Hylastes cunicularius* | x |  |  |  |  |  |  | x | x |  |  | 1661 | 72 |
| *Hylastes opacus* | x |  |  |  |  |  |  | x | x |  |  | 7 | 0 |
| *Hylecoetus dermestoides* |  |  | x |  |  | x |  | x | x |  |  | 199 | 0 |
| *Hylobius abietis* | x |  |  |  |  | x |  | x | x |  |  | 4 | 7 |
| *Hylobius piceus* | x |  |  |  |  |  |  | x | x |  |  | 1 | 0 |
| *Hylurgops glabratus* | x |  |  |  |  |  |  | x | x |  |  | 141 | 0 |
| *Hylurgops palliatus* | x |  |  |  |  |  |  | x | x |  |  | 7 | 0 |
| *Ips typographus* | x |  |  |  |  |  |  | x | x |  |  | 5 | 0 |
| *Judolia sexmaculata* | x |  |  |  |  | x |  | x | x |  |  | 24 | 0 |
| *Latridius hirtus* |  |  | x |  |  |  |  | x | x |  |  | 35 | 1 |
| *Leptusa fumida* |  |  | o |  |  |  |  | x | x |  |  | 30 | 25 |
| *Leptusa norvegica* |  |  | o |  |  |  |  | x | x |  |  | 1 | 0 |
| *Leptusa pulchella* |  |  | o |  |  |  |  | x | x |  |  | 91 | 78 |
| *Lordithon speciosus* |  |  |  |  | x |  |  | x | x |  |  | 51 | 4 |
| *Lordithon trimaculatus* |  |  |  |  | x |  |  | x | x |  |  | 9 | 0 |
| *Lygistopterus sanguineus* |  |  |  |  | x |  |  | x | x |  |  | 3 | 2 |
| *Magdalis phlegmatica* |  |  |  |  |  | x |  | x | x |  |  | 1 | 0 |
| *Magdalis violacea* | x |  |  |  |  |  |  | x | x |  |  | 1 | 0 |
| *Malthodes brevicollis* |  |  |  | x | x |  |  | x | x |  |  | 1 | 1 |
| *Malthodes flavoguttatus* |  |  |  |  | x |  |  | x | x |  |  | 1 | 0 |
| *Malthodes fuscus* |  |  |  |  | x |  |  | x | x |  |  | 3 | 35 |
| *Malthodes guttifer* |  |  |  |  | x |  |  | x | x |  |  | 1 | 49 |
| *Malthodes marginatus* |  |  |  |  | x |  |  | x | x |  |  | 1 | 0 |
| *Melanotus castanipes* |  |  |  |  | x | x |  | x | x |  |  | 614 | 8 |
| *Microscydmus minimus* |  |  |  |  | x |  |  | x | x |  |  | 11 | 0 |
| *Molorchus minor* | x |  |  |  |  | x |  | x | x |  |  | 17 | 5 |
| *Mordella holomelaena* |  |  | x |  |  |  |  | x | x |  |  | 4 | 0 |
| *Mycetochara flavipes* |  |  |  |  |  |  |  | x | x |  |  | 6 | 0 |
| *Mycetophagus multipunctatus* |  |  | x |  |  |  |  | x | x |  |  | 2 | 0 |
| *Mycetophagus populi* |  |  | x |  |  |  |  | x | x |  |  | 11 | 0 |
| *Nudobius lentus* |  |  |  |  | x |  |  | x | x |  |  | 8 | 4 |
| *Octotemnus glabriculus* |  |  | x |  |  |  |  | x | x |  |  | 4 | 0 |
| *Orchesia micans* |  |  | x |  |  |  |  | x | x |  |  | 24 | 0 |
| *Orthocis alni* |  |  | x |  |  |  |  | x | x |  |  | 57 | 0 |
| *Orthocis festivus* |  |  | x |  |  |  |  | x | x |  |  | 1 | 0 |
| *Orthocis linearis* |  |  | x |  |  |  |  | x | x |  |  | 2 | 0 |
| *Orthoperus nigrescens* |  |  | x |  |  |  |  | x | x |  |  | 3 | 0 |
| *Orthotomicus laricis* | x |  |  |  |  |  |  | x | x |  |  | 2 | 1 |
| *Orthotomicus proximus* | x |  |  |  |  |  |  | x | x |  |  | 1 | 0 |
| *Orthotomicus suturalis* | x |  |  |  |  |  |  | x | x |  |  | 4 | 2 |
| *Ostoma ferruginea* |  |  | x |  |  |  |  | x | x |  |  | 60 | 9 |
| *Oxymirus cursor* |  |  |  |  |  | x |  | x | x |  |  | 12 | 2 |
| *Pediacus fuscus* |  |  | x |  |  |  |  | x | x |  |  | 1 | 3 |
| *Phloeonomus planus* |  |  |  |  | o |  |  | x | x |  |  | 13 | 6 |
| *Phloeonomus sjoebergi* |  |  |  |  | o |  |  | x | x |  |  | 163 | 10 |
| *Phloeopora concolor* |  |  |  |  | x |  |  | x | x |  |  | 9 | 1 |
| *Phloeopora corticalis* |  |  |  |  | o |  |  | x | x |  |  | 13 | 0 |
| *Phloeopora testacea* |  |  |  |  | o |  |  | x | x |  |  | 6 | 0 |
| *Phloeotribus spinulosus* | x |  |  |  |  |  |  | x | x |  |  | 46 | 0 |
| *Phyllodrepa linearis* |  |  | o |  | o |  |  | x | x |  |  | 45 | 33 |
| *Phyllodrepa melanocephala* |  |  | o |  | o |  |  | x | x |  |  | 43 | 1 |
| *Pissodes gyllenhalii* | x |  |  |  |  |  |  | x | x |  |  | 1 | 0 |
| *Pissodes harcyniae* | x |  |  |  |  |  |  | x | x |  |  | 2 | 0 |
| *Pissodes pini* | x |  |  |  |  |  |  | x | x |  |  | 1 | 0 |
| *Pissodes piniphilus* | x |  |  |  |  |  |  | x | x |  |  | 1 | 0 |
| *Pityogenes bidentatus* | x |  |  |  |  |  |  | x | x |  |  | 21 | 0 |
| *Pityogenes chalcographus* | x |  |  |  |  |  |  | x | x |  |  | 248 | 2 |
| *Pityophagus ferrugineus* |  |  |  |  | x |  |  | x | x |  |  | 12 | 0 |
| *Pityophthorus micrographus* | x |  |  |  |  |  |  | x | x |  |  | 2 | 0 |
| *Placusa atrata* |  |  |  |  | o |  |  | x | x |  |  | 8 | 2 |
| *Placusa tachyporoides* |  |  |  |  | o |  |  | x | x |  |  | 7 | 0 |
| *Platycerus caprea* |  |  |  |  |  | x |  | x | x |  |  | 42 | 0 |
| *Platysoma angustatum* |  |  |  |  | x |  |  | x | x |  |  | 2 | 0 |
| *Platystomus albinus* |  |  |  |  |  | x |  | x | x |  |  | 3 | 0 |
| *Plegaderus vulneratus* |  |  |  |  | x |  |  | x | x |  |  | 8 | 7 |
| *Pogonocherus decoratus* | x |  |  |  |  | x |  | x | x |  |  | 2 | 0 |
| *Pogonocherus fasciculatus* | x |  |  |  |  | x |  | x | x |  |  | 11 | 0 |
| *Polygraphus poligraphus* | x |  |  |  |  |  |  | x | x |  |  | 409 | 0 |
| *Polygraphus punctifrons* | x |  |  |  |  |  |  | x | x |  |  | 341 | 25 |
| *Polygraphus subopacus* | x |  |  |  |  |  |  | x | x |  |  | 85 | 1 |
| *Pteryngium crenatum* |  |  | x |  |  |  |  | x | x |  |  | 20 | 16 |
| *Pteryx suturalis* |  |  | x |  |  |  |  | x | x |  |  | 387 | 8 |
| *Ptilinus fuscus* |  |  |  |  |  | x |  | x | x |  |  | 4 | 0 |
| *Ptiliolum caledonicum* |  |  | x |  |  |  |  | x | x |  |  | 4 | 0 |
| *Ptinella aptera* |  |  | x |  |  |  |  | x | x |  |  | 1 | 0 |
| *Ptinella johnsoni* |  |  | x |  |  |  |  | x | x |  |  | 2 | 0 |
| *Ptinella tenella* |  |  | x |  |  |  |  | x | x |  |  | 1 | 0 |
| *Ptinus subpilosus* | x |  |  |  |  |  |  | x | x |  |  | 21 | 0 |
| *Pyropterus nigroruber* |  |  |  |  | x |  |  | x | x |  |  | 1 | 0 |
| *Pytho depressus* | x |  |  |  |  |  |  | x | x |  |  | 4 | 0 |
| *Quedius plagiatus* |  |  |  |  | x |  |  | x | x |  |  | 235 | 196 |
| *Rabocerus foveolatus* |  |  |  |  | x |  |  | x | x |  |  | 45 | 0 |
| *Rabocerus gabrieli* |  |  |  |  | x |  |  | x | x |  |  | 25 | 0 |
| *Rhagium inquisitor* | x |  |  |  |  | x |  | x | x |  |  | 7 | 2 |
| *Rhagium mordax* | x |  |  |  |  |  |  | x | x |  |  | 24 | 0 |
| *Rhizophagus ferrugineus* |  |  |  |  | x |  |  | x | x |  |  | 51 | 0 |
| *Rhizophagus nitidulus* |  |  |  |  | x |  |  | x | x |  |  | 65 | 16 |
| *Rhizophagus parvulus* |  |  |  |  | x |  |  | x | x |  |  | 468 | 9 |
| *Rhyncolus ater* |  |  |  |  |  | x |  | x | x |  |  | 1 | 199 |
| *Rhyncolus sculpturatus* |  |  |  |  |  | x |  | x | x |  |  | 22 | 7 |
| *Ropalodontus strandi* |  |  | x |  |  |  |  | x | x |  |  | 1 | 0 |
| *Salpingus planirostris* |  |  |  |  | x |  |  | x | x |  |  | 3 | 0 |
| *Salpingus ruficollis* |  |  |  |  | x |  |  | x | x |  |  | 73 | 1 |
| *Scaphisoma subalpinum* |  |  | o |  |  |  |  | x | x |  |  | 16 | 0 |
| *Schizotus pectinicornis* | x |  |  |  |  |  |  | x | x |  |  | 8 | 0 |
| *Scolytus ratzeburgi* | x |  |  |  |  |  |  | x | x |  |  | 8 | 0 |
| *Scydmoraphes minutus* |  |  |  |  | x |  |  | x | x |  |  | 1 | 1 |
| *Serropalpus barbatus* |  |  |  |  |  | x |  | x | x |  |  | 1 | 0 |
| *Silvanoprus fagi* |  |  |  |  | o |  |  | x | x |  |  | 18 | 0 |
| *Silvanus bidentatus* |  |  |  |  | o |  |  | x | x |  |  | 1 | 0 |
| *Soronia grisea* |  | x |  |  |  |  |  | x | x |  |  | 2 | 0 |
| *Soronia punctatissima* |  | x | x |  |  |  |  | x | x |  |  | 4 | 0 |
| *Sphaeriestes bimaculatus* |  |  |  |  | x |  |  | x | x |  |  | 3 | 0 |
| *Stenotrachelus aeneus* | x |  |  |  |  | x |  | x | x |  |  | 14 | 0 |
| *Sulcacis affinis* |  |  | x |  |  |  |  | x | x |  |  | 2 | 0 |
| *Sulcacis fronticornis* |  |  | x |  |  |  |  | x | x |  |  | 3 | 0 |
| *Synchita humeralis* |  | x | x |  |  |  |  | x | x |  |  | 4 | 0 |
| *Tetratoma ancora* |  |  | x |  |  |  |  | x | x |  |  | 71 | 0 |
| *Tetropium castaneum* | x |  |  |  |  |  |  | x | x |  |  | 17 | 0 |
| *Tetropium fuscum* | x |  |  |  |  |  |  | x | x |  |  | 16 | 0 |
| *Thanasimus femoralis* |  |  |  |  | x |  |  | x | x |  |  | 4 | 0 |
| *Thanasimus formicarius* |  |  |  |  | x |  |  | x | x |  |  | 4 | 0 |
| *Tomoxia bucephala* |  |  |  |  |  | x |  | x | x |  |  | 2 | 0 |
| *Trichius fasciatus* |  |  |  |  |  | x |  | x | x |  |  | 5 | 0 |
| *Triplax aenea* |  |  | x |  |  |  |  | x | x |  |  | 68 | 0 |
| *Triplax russica* |  |  | x |  |  |  |  | x | x |  |  | 209 | 0 |
| *Triplax scutellaris* |  |  | x |  |  |  |  | x | x |  |  | 131 | 0 |
| *Trypodendron domesticum* |  |  | x |  |  |  |  | x | x |  |  | 12 | 0 |
| *Trypodendron laeve* |  |  | x |  |  |  |  | x | x |  |  | 1 | 0 |
| *Trypodendron lineatum* |  |  | x |  |  |  |  | x | x |  |  | 163 | 0 |
| *Trypodendron signatum* |  |  | x |  |  |  |  | x | x |  |  | 189 | 0 |
| *Trypophloeus bispinulus* | x |  |  |  |  |  |  | x | x |  |  | 5 | 0 |
| *Xylechinus pilosus* | x |  |  |  |  |  |  | x | x |  |  | 448 | 0 |
| *Xylita laevigata* |  |  | x |  |  |  |  | x | x |  |  | 289 | 5 |
| *Xylotrechus rusticus* | x |  |  |  |  | x |  | x | x |  |  | 5 | 0 |
| *Phryganophilus ruficollis* |  |  |  |  | o | o | x |  | x |  |  | 1 | 0 |
| *Ampedus nigroflavus* |  |  |  |  | x | x | x |  | x |  |  | 1 | 0 |
| *Atomaria affinis* |  |  | x |  |  |  | x |  | x |  |  | 1 | 0 |
| *Atomaria alpina* |  |  | x |  |  |  | x |  | x |  |  | 51 | 7 |
| *Atomaria badia* |  |  | x |  |  |  | x |  | x |  |  | 1 | 2 |
| *Atomaria lapponica* |  |  | x |  |  |  | x |  | x |  |  | 1 | 7 |
| *Cerylon deplanatum* |  |  | x |  |  |  | x |  | x |  |  | 7 | 0 |
| *Cis dentatus* |  |  | x |  |  |  | x |  | x |  |  | 37 | 2 |
| *Cis quadridens* |  |  | x |  |  |  | x |  | x |  |  | 7 | 0 |
| *Corticaria polypori* |  |  | x |  |  |  | x |  | x |  |  | 7 | 0 |
| *Denticollis borealis* |  |  |  |  | x | x | x |  | x |  |  | 8 | 0 |
| *Enicmus apicalis* |  |  | x |  |  |  | x |  | x |  |  | 34 | 2 |
| *Enicmus planipennis* |  |  | x |  |  |  | x |  | x |  |  | 12 | 0 |
| *Ennearthron laricinum* |  |  | x |  |  |  | x |  | x |  |  | 19 | 5 |
| *Eudectus giraudi* |  |  | o |  |  |  | x |  | x |  |  | 100 | 1 |
| *Euryusa castanoptera* |  |  | o |  |  |  | x |  | x |  |  | 48 | 1 |
| *Evodinus borealis* | x |  |  |  |  |  | x |  | x |  |  | 2 | 0 |
| *Hallomenus axillaris* |  |  | x |  |  |  | x |  | x |  |  | 7 | 0 |
| *Harminius undulatus* |  |  |  |  | x | x | x |  | x |  |  | 12 | 11 |
| *Lacon conspersus* |  |  |  |  | x |  | x |  | x |  |  | 4 | 1 |
| *Lacon fasciatus* |  |  |  |  | x |  | x |  | x |  |  | 34 | 16 |
| *Leiestes seminigra* |  |  | x |  |  |  | x |  | x |  |  | 5 | 0 |
| *Mycetochara obscura* |  |  |  |  |  |  | x |  | x |  |  | 23 | 0 |
| *Mycetophagus fulvicollis* |  |  | x |  |  |  | x |  | x |  |  | 20 | 1 |
| *Olisthaerus megacephalus* |  |  |  |  | o |  | x |  | x |  |  | 2 | 0 |
| *Orchesia fasciata* |  |  | x |  |  |  | x |  | x |  |  | 11 | 0 |
| *Orchesia minor* |  |  | x |  |  |  | x |  | x |  |  | 3 | 0 |
| *Pentanota meuseli* |  |  |  |  | o |  | x |  | x |  |  | 1 | 0 |
| *Platysoma minus* |  |  |  |  | x |  | x |  | x |  |  | 1 | 5 |
| *Stagetus borealis* |  |  | x |  |  | x | x |  | x |  |  | 2 | 0 |
| *Tachyta nana* |  |  |  |  | x |  | x |  | x |  |  | 6 | 0 |
| *Zilora ferruginea* |  |  | x |  |  |  | x |  | x |  |  | 5 | 14 |
| *Lasconotus jelskii* |  |  |  |  | x |  | x |  | x |  |  | 1 | 6 |
| *Melandrya dubia* |  |  | o |  |  |  | x |  | x |  |  | 1 | 0 |
| *Phymatura brevicollis* |  |  |  |  | o |  | x |  | x |  |  | 1 | 0 |
| *Atheta sg Dimetrota picipennoides* |  | o | o |  | x |  |  | x |  |  |  | 2 | 0 |
| *Agathidium arcticum* |  |  | x |  |  |  |  | x |  | x |  | 0 | 3 |
| *Amidobia talpa* |  |  |  |  |  |  |  | x |  |  |  | 13 | 0 |
| *Anopleta corvina* |  |  |  |  | o |  |  | x |  | x |  | 0 | 1 |
| *Aphodius tenellus* |  |  |  |  |  |  |  | x |  |  |  | 1 | 0 |
| *Archarius salicivorus* |  |  |  |  |  |  |  | x |  |  |  | 1 | 0 |
| *Atheta sg Atheta allocera* |  | o | o |  | x |  |  | x |  | x |  | 0 | 1 |
| *Atomaria procerula* |  |  | x |  |  |  |  | x |  | x |  | 0 | 2 |
| *Atomaria zetterstedti* |  |  | x |  |  |  |  | x |  |  | x | 0 | 1 |
| *Bius thoracicus* |  |  | x |  |  |  |  | x | x |  |  | 0 | 1 |
| *Byturus aestivus* |  |  |  |  |  |  |  | x |  |  |  | 1 | 0 |
| *Callidium coriaceum* | x |  |  |  |  | x |  | x | x |  |  | 0 | 5 |
| *Cerylon sp.* |  |  |  |  |  |  |  |  |  |  |  | 0 | 4 |
| *Cis sp.* |  |  |  |  |  |  |  |  |  |  |  | 0 | 1 |
| *Corticaria foveola* |  |  | x |  |  |  |  | x |  | x |  | 0 | 4 |
| *Corticaria longicollis* |  |  | x |  |  |  |  | x |  | x |  | 0 | 10 |
| *Corticarina latipennis* |  |  | x |  |  |  |  | x |  | x |  | 0 | 1 |
| *Corticarina similata* |  |  | x |  |  |  |  | x |  | x |  | 0 | 2 |
| *Cryptophagus affinis* |  |  |  |  |  |  |  | x |  |  |  | 1 | 0 |
| *Dalotia coriaria* |  |  |  |  | o |  |  | x |  | x |  | 0 | 1 |
| *Dermestes lardarius* |  | x |  |  |  |  |  | x |  |  | x | 0 | 1 |
| *Dienerella filum* |  |  | x |  |  |  |  | x |  | x |  | 0 | 28 |
| *Malthinus punctatus* |  |  |  | x | x |  |  | x | x |  |  | 0 | 1 |
| *Microdota wireni* |  |  |  |  |  |  |  | x |  |  |  | 2 | 0 |
| *Nepachys cardiacae* |  |  |  |  | x |  |  | x | x |  |  | 0 | 7 |
| *Olisthaerus substriatus* |  |  |  |  | o |  | x |  | x |  |  | 0 | 6 |
| *Omalium caesum* |  |  |  |  | o |  |  | x |  | x |  | 0 | 2 |
| *Phloeonomus punctipennis* |  |  |  |  | o |  |  | x | x |  |  | 0 | 1 |
| *Phloeonomus pusillus* |  |  |  |  | x |  |  | x | x |  |  | 0 | 14 |
| *Phosphuga atrata* |  |  |  |  | x |  |  | x |  | x |  | 0 | 1 |
| *Phytobius velaris* |  |  |  |  |  |  |  | x |  |  |  | 1 | 0 |
| *Placusa depressa* |  |  |  |  | o |  |  | x | x |  |  | 0 | 1 |
| *Pteryx splendens* |  |  | x |  |  |  |  | x | x |  |  | 0 | 1 |
| *Quedius maurus* |  |  |  |  | o |  |  | x | x |  |  | 0 | 2 |
| *Sogda perrisii* |  |  |  |  |  |  |  | x |  |  |  | 1 | 0 |
| *Thiasophila angulata* |  | o |  |  | o |  |  | x |  |  | x | 3 | 0 |
| *Thiasophila wockii* |  |  |  |  | o |  |  | x | x |  |  | 26 | 1 |
| *Xantholinus tricolor* |  |  |  |  | o |  |  | x |  | x |  | 0 | 3 |
